# Comparing co-evolution methods and their application to template-free protein structure prediction

**DOI:** 10.1093/bioinformatics/btw618

**Published:** 2016-09-27

**Authors:** Saulo Henrique Pires de Oliveira, Jiye Shi, Charlotte M Deane

**Affiliations:** 1 Department of Statistics, University of Oxford, Oxford, UK; 2 Department of Informatics, UCB Pharma, Slough, UK; 3 Shanghai Institute of Applied Physics, Chinese Academy of Sciences, Shanghai, China

## Abstract

**Motivation:**

Co-evolution methods have been used as contact predictors to identify pairs of residues that share spatial proximity. Such contact predictors have been compared in terms of the precision of their predictions, but there is no study that compares their usefulness to model generation.

**Results:**

We compared eight different co-evolution methods for a set of ∼3500 proteins and found that metaPSICOV stage 2 produces, on average, the most precise predictions. Precision of all the methods is dependent on SCOP class, with most methods predicting contacts in all α and membrane proteins poorly. The contact predictions were then used to assist in *de novo* model generation. We found that it was not the method with the highest average precision, but rather metaPSICOV stage 1 predictions that consistently led to the best models being produced. Our modelling results show a correlation between the proportion of predicted long range contacts that are satisfied on a model and its quality. We used this proportion to effectively classify models as correct/incorrect; discarding decoys classified as incorrect led to an enrichment in the proportion of good decoys in our final ensemble by a factor of seven. For 17 out of the 18 cases where correct answers were generated, the best models were not discarded by this approach. We were also able to identify eight cases where no correct decoy had been generated.

**Availability and Implementation:**

Data is available for download from: http://opig.stats.ox.ac.uk/resources.

**Supplementary information:**

Supplementary data are available at *Bioinformatics* online.

## 1 Introduction

*De novo* protein structure prediction, also known as template-free modelling, is the method of choice for structure prediction when structural homologs to a target sequence cannot be effectively identified (Moult *et al.*, 2014).

Recently, it has been shown that the use of predicted contacts can significantly improve *de novo* protein structure prediction, (e.g. Braun *et al.*, 2015; Hopf *et al.*, 2012, 2015; Jones *et al.*, 2012, 2014; Kamisetty *et al.*, 2013; Kim *et al.*, 2014; Marks *et al.*, 2011, 2012; Ovchinnikov *et al.*, 2015; Seemayer *et al.*, 2014). Such contacts tend to be predicted based on the principle of correlated mutations. Under this theory, sequence positions in a multiple sequence alignment that mutate in a correlated fashion are likely to represent residues that share a spatial proximity (Göbel *et al.*, 1994; Pazos *et al.*, 1997).

The notion of correlated mutations was first introduced by Göbel *et al.* (1994), in which a simplistic formulation of correlated mutations was used to infer spatial constraints from a multiple sequence alignment. The precision of contacts inferred from correlated mutations has recently been improved with the exponential growth in the number of protein sequences available in the public domain and the emergence of new statistical techniques.

Mean Field Direct Coupling Analysis (mfDCA) was one of the first of these new techniques (Morcos *et al.*, 2011). mfDCA uses the information of all columns in the alignment when ascertaining the correlation between two individual columns. An implementation of this method, EV-Fold, was initially tested on 15 soluble proteins and shown to obtain a predicted contact precision greater than 50% for 12 cases (Marks *et al.*, 2011). Marks and colleagues also reported how EV-Fold could be used to improve *de novo* structure prediction both for soluble (Marks *et al.*, 2011, 2012) and transmembrane proteins (Hopf *et al.*, 2012). Another approach uses an estimate of the inverse covariance matrix to assign a score to residue pairs. This approach has been implemented in PSICOV (Jones *et al.*, 2012) and used to predict protein contacts for 150 targets. The top-L/5 long-range contact predictions (sequence separation >23) for a protein of length *L* were shown to have a precision greater than 0.5. These contact predictions were then used in the fragment-assembly software FRAGFOLD (Kosciolek and Jones, 2014), generating accurate models for 100 of the 150 targets. PSICOV predictions were also used with FILM3 to assist in protein structure prediction for 28 membrane proteins, producing accurate models for 26 of these proteins (Nugent and Jones, 2013).

A third approach has been developed based on learning the direct couplings as parameters of a Probabilistic Graphical Model (Markov random field) by maximizing its pseudo-likelihood. This approach has several implementations, including plmDCA (Ekeberg *et al.*, 2013), GREMLIN (Kamisetty *et al.*, 2013) and CCMPred (Seemayer *et al.*, 2014). GREMLIN has been tested on 329 protein targets selected from the Continuous, Automated Model Evaluation (CAMEO) (Haas *et al.*, 2013) and shown to outperform PSICOV, plmDCA and mfDCA. Another tool, EPC-map (Schneider and Brock, 2014), combines contact predictions output by GREMLIN with physicochemical information, reporting an average improvement in precision of 4.4% compared to GREMLIN.

The three contact prediction approaches described above have been shown to be partially orthogonal and output a significant number of non-overlapping predictions (Monastyrskyy *et al.*, 2015). Meta-predictors have been developed to combine the methods in an attempt to produce a consensus. PconsC (Skwark *et al.*, 2013) and PconsC2 (Skwark *et al.*, 2014) were based on using sixteen sets of predictions output by PSICOV and plmDCA, each obtained using a different multiple sequence alignment. A machine learning approach generates the consensus set of predictions. PconsC2 was shown to obtain higher precision when compared to PSICOV, plmDCA and PconsC on three different sets. A second consensus method, metaPSICOV, uses predictions output by Freecontact (a mfDCA implementation) (Kaján *et al.*, 2014), PSICOV and CCMPred. Resulting contact predictions are used as features in a two-layer neural network.

Specialist predictors like Bbcontacts (Andreani and Söding, 2015) have been developed, in this case, to infer only beta-strand pairing based on the output of CCMPred. It has been shown to achieve 50% precision for β–β residue pairs at 50% recall using predicted secondary structure.

It has been suggested that the number of sequences required to produce reliable contact predictions should be of the order of 5×L, where *L* is the length of the protein. A recent study (Kamisetty *et al.*, 2013) estimated that the number of protein families in Pfam for which no homologue structure is known (5146) and for which there are more than 5×L sequences in the multiple sequence alignment is 291. According to the authors, only about 15% of *de novo* modelling cases can benefit from predicted contact information.

Different contact prediction software has been tested with different structure prediction programs and on different structure test sets. It is, therefore, hard to draw an unbiased comparison between different contact predictors in order to identify the one that is most suitable for *de novo* protein structure prediction. It is also necessary to ascertain how we can use such contact predictions most efficiently in order to improve contact assisted *de novo* protein structure prediction. In order to try and answer these questions, here we have tested several different co-evolution methods generating more than 3 million decoys, using approximately a decade of CPU core-hours.

## 2 Methods

### 2.1 Contact definitions

Two protein residues are defined to be in contact if their C-βs (C-αs for Glycine) are less than 8 Å apart (Marks *et al.*, 2011). Trivial contacts occur due to residues being less than five residues apart and are not considered in the scope of our analyses. A short-range contact between residues *i* and *j* is defined when 5≤|i−j|≤23. A long-range contact is defined when |i−j|>23 (Jones *et al.*, 2012). We also define six classes of contacts between different secondary structure types: loop–loop as formed between two loop residues, loop–helix, loop–strand, helix–helix, helix–strand and strand–strand.

### 2.2 Generation of multiple sequence alignments

Generating input alignments has been identified as a crucial step for predicting contacts (Feinauer *et al.*, 2014). To ensure consistency in our analyses, the same alignment has been used as input for all contact predictors, the exception being PConsC2 (for more details, refer to Section 2.3). The multiple sequence alignment (MSA) was computed according to the following parameters:

- HHBlits version 2.0.15 June 2012
- Database: Uniprot20_2013_03
- Iterations: 3
- Maximum Pairwise Sequence Identity: 99%
- Minimum Coverage with master sequence: 60%
- Maxfilt: 500000
- Diff: inf

### 2.3 Contact prediction methods

Mean Field Direct Coupling Analysis (mfDCA) is a class of contact predictors based on a maximum entropy model. For the analyses described in this work, we used both Freecontact version 1.0.21, with standard parameters (Kaján *et al.*, 2014) and PSICOV version 2.1 with a target precision matrix sparsity of 0.03 and default parameters (Jones *et al.*, 2012).

MfDCA and PSICOV are significantly different in terms of their derivation and estimation procedures. However, they both rely on estimating the inverse of the covariance matrix, which follows from their maximum entropy model. The other category of contact predictors avoids the approximation of the inverse covariance matrix altogether by maximizing a pseudo-likelihood. For the analyses described in this work, we have used two implementations of plmDCA: CCMPred v0.1.0 with standard parameters (Seemayer *et al.*, 2014) and GREMLIN v2.01 with standard parameters (Kamisetty *et al.*, 2013).

PConsC uses different sets of contact predictions output by PSICOV and plmDCA (standard parameters are used for both methods). In total, eight different MSAs are used as input for PSICOV and plmDCA, resulting in sixteen sets of predicted contacts. These MSAs are generated using HHblits (Remmert *et al.*, 2012) against its bundled nr20 database or Jackhmmer (Johnson *et al.*, 2010) against UniRef100, for four different E-value cut-offs ($10^{-40},\;10^{-10},\;10^{-4}$, 1). PConsC uses a random forest method to perform the classification of the sets of predictions. PConsC2 enriches the predictions obtained by PConsC by means of a deep-learning method, which is based on the notion that protein contacts are likely to be in proximity to other protein contacts in the contact map. We have used PConsC2 with standard parameters (Skwark *et al.*, 2014) to perform predictions on a smaller dataset of 41 proteins, but the tool failed to produce an output for approximately 25% of the cases. PConsC2 requires 16 multiple sequence alignments as input and these were generated using the default MSA protocol described in (Skwark *et al.*, 2014).Given the time taken to generate 16 multiple sequence alignments and the likely failure rate, we decided not to use PConsC2 to perform predictions for our larger dataset of 3458 proteins.

MetaPSICOV is a meta-predictor based on contact predictions output by Freecontact (v1.0.21), PSICOV (v2.1) and CCMPred (v0.1.0). Unlike PConsC/PConsC2, in metaPSICOV the same alignment is used for all three methods.

MetaPSICOV outputs predictions from two stages in its procedure. The prediction from the second stage is an enhancement of the first stage and is reported to be more precise (Jones *et al.*, 2014). However, predictions from metaPSICOV Stage 1 were shown to yield better modelling results (Jones *et al.*, 2014). MetaPSICOV is also capable of outputting predictions for pairs of residues forming a backbone hydrogen bond, which is referred to as metaPSICOV-HB. In the analyses described in this chapter, we have used metaPSICOV v1.01 with standard parameters (Jones *et al.*, 2014).

Bbcontacts uses predicted contact maps as output by CCMPred to identify contacts between β-strands. We have used CCMPred v0.1.0 (Seemayer *et al.*, 2014) to generate predicted contact maps and used Bbcontacts with standard parameters to infer beta-strand contacts (Andreani and Söding, 2015).

### 2.4 Number of predictions considered

For the sake of establishing a fair comparison between all methods, we have considered only up to *L* predicted contacts in our analyses. The software bbcontacts usually outputs less than L/2 predicted contacts so its precision and spread were computed based on the number of predictions available.

### 2.5 Data sets

For the comparison of the precision of different contact predictors, we have used a test set comprised of 3458 proteins. These proteins were selected randomly from the Astral 2.05 database (PDB SCOPe 40% ID) (Chandonia, *et al.*, 2004). This dataset contains at least one member of 1668 distinct SCOP folds and each of these folds is represented by 1.95 sequences, on average. Given that model generation is a computationally intensive task, we have selected a smaller set of 41 proteins for which structure prediction was carried out (SI-Table 1—PDB-Representative dataset). This dataset was constructed to be representative of the proportions of proteins in each SCOP class and across different lengths observed in the PDB. We manually curated the PDB to choose structures that described continuous, well resolved (resolution less than 2.5 Å), single-chain proteins. For each length range, a representative structure that fulfilled these criteria was chosen. Care was also taken to select proteins belonging to different Pfam families.

### 2.6 Validation metrics

The **precision** of predicted contacts (also referred to as PPV) is the percentage of true positives in the predictions output for a given target.

The **spread of correct predicted contacts** is defined as the largest protein segment for which no correct contacts were predicted. The spread is shown as a proportion of the protein length and it measures the largest portion of the target structure for which no correct contact information is available. This allows us to discern between a set of predictions that is restricted to a portion of the target structure and a set of predictions that is evenly spread across the protein’s length.

### 2.7 Model generation

The contact predictions output by PSICOV, Freecontact, CCMPred, Bbcontacts, metaPSICOV Stage 1, metaPSICOV Stage 2 and metaPSICOV HB were used as input for our cotranslational template-free structure prediction method SAINT2. An outline of our fragment-based *de novo* structure predictor SAINT2 is given in Supplementary Text S1.

We generated 10 000 decoys for each set of predicted contacts and for all targets in our PDB-Representative dataset. Standard parameters and scoring weights were used during decoy generation (for more information, refer to Supplementary Text S1). A decoy was considered to be correct if its TM-Score (Zhang and Skolnick, 2004) was greater than 0.5, following (Xu and Zhang, 2010).

## 3 Results

### 3.1 Comparing nine contact predictors

**Fig. 1:**
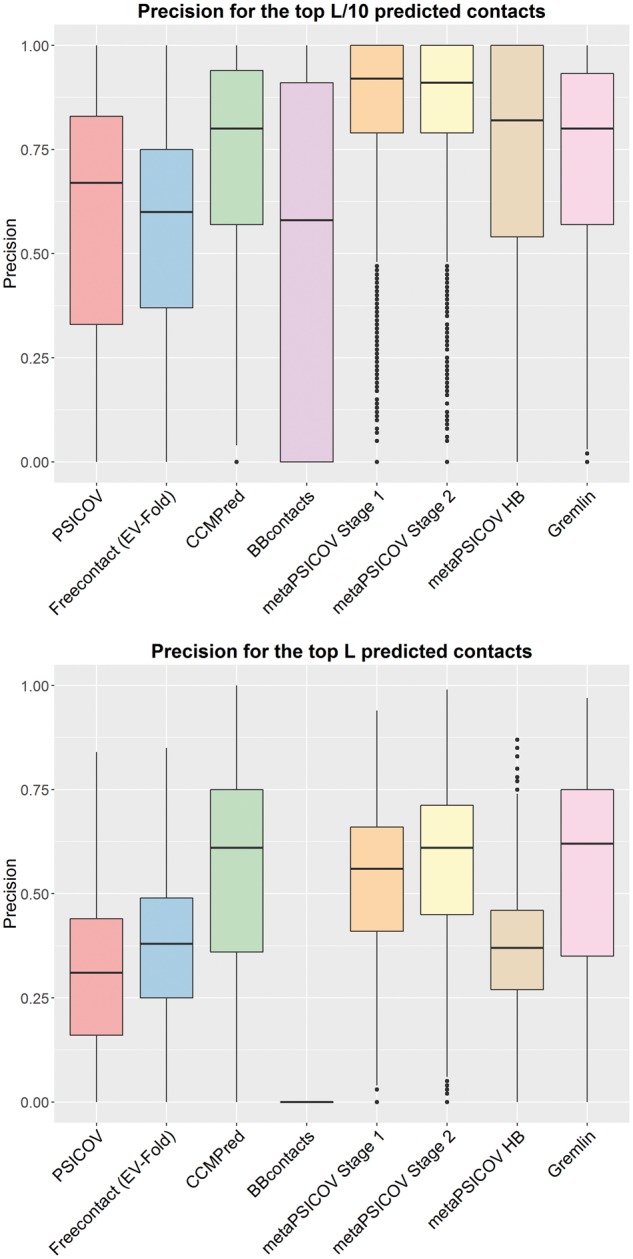
Precision of eight contact predictors for 3458 proteins in our Astral dataset. Precision is shown for the top L/10 predicted contacts (top) and top *L* predicted contacts (bottom). Precision for top L/5 and top L/2 predicted contacts can be seen in SI Figure S2. A comparison including the meta-predictor PConsC2 for a smaller data-set of 32 proteins in shown in SI Figure S3

We tested eight state-of-the-art contact prediction methods (FreeContact, PSICOV, CCMPred, Bbcontacts, metaPSICOV stage 1, metaPSICOV stage2, metaPSICOV HB and GREMLIN) on the 3458 proteins in our Astral dataset. We compared the precision of the methods for the top L/10, L/5, L/2 and *L* predicted contacts (Fig. 1 and Supplementary Fig. S2), where *L* is the length of the protein. Our results show that contact predictions output by metaPSICOV Stage 2 are, on average, the most precise for the top L/10, L/5 and L/2 predicted contacts. CCMPred, GREMLIN and metaPSICOV Stage 2 are, on average, comparable for the top *L* predictions. When considering the top *L* predictions, metaPSICOV stage 2 achieved more than 50% precision for 2358 cases (68.2%), whereas CCMPred and GREMLIN achieved more than 50% precision for 2195 (63.5%) and 2192 (63.4%), respectively. A precision of at least 50% has been used to determine if a set of contact predictions can be useful for model generation (Jones *et al.*, 2012).

We have assessed the overlap between metaPSICOVs training set and our Astral dataset. We found that 54 out of the 624 proteins used to train metaPSICOV were also present among the 3458 proteins in our dataset. Furthermore, we encountered 112 distinct SCOP folds that were represented in both sets, corresponding to less than 10% of the unique folds described by our Astral dataset. Removal of overlapping proteins and folds did not lead to significant changes on the precision observed for metaPSICOV compared to other methods.

We have also performed a comparison including the predictor PConsC2 for the 41 proteins in our PDB-Representative dataset. PConsC2 failed to produce an output for nine cases. The results for the remaining 32 cases can be seen in Supplementary Figures S3 and S4, where metaPSICOV stage 2 still presented the highest average precision for the top L/10, L/5, L/2 predicted contacts. For this dataset, the precision of PConsC2 and metaPSICOV stage 2 for the top *L* predictions were comparable.

Long-range contacts have been suggested to be more useful than short-range contacts for *de novo* protein structure prediction (Jones *et al.*, 2012). Here, we define a long-range contact as one between two residues that are more than 23 residues apart. We have quantified the precision and the proportion of short and long-range predicted contacts generated in our Astral dataset (Supplementary Fig. S5). While CCMPred, GREMLIN and metaPSICOV stages 2 achieved similar average precisions for both short and long-range predictions, metaPSICOV stage 1 and 2 are more consistent in producing good predictions. For short range contacts, metaPSICOV stage 2 produced predictions with at least 50% precision for 69.2% of the cases compared to 64.1% (CCMPred) and 64.4% (GREMLIN). For long range contacts, metaPSICOV stage 1 produced predictions with at least 50% precision for 65.2% of the cases compared to 58.9% (CCMPred) and 58.8% (GREMLIN).

It has been reported that the precision of predicted contacts is dependent on protein length and on the number of available sequences (Ovchinnikov *et al.*, 2015). We assessed the effect of the number of sequences in the MSA on the precision of contacts predicted by the eight different contact predictors. Since Bbcontacts did not generate L/2 predictions for more than 50% of the cases, data for this method has been omitted. We used the predictions generated by the seven remaining methods to build a confidence interval for the number of sequences necessary to achieve a precision of at least 50% for generated predictions (Fig. 3). We performed a linear regression using the number of effective sequences (Neff) as a dependent variable and using the precision of each predictor as an independent variable. We then use our linear model to compute the confidence interval for Neff assuming an observed precision of 50%. We have used the definition of Neff in (Kamisetty *et al.*, 2013). Our results show that metaPSICOV Stage 2 is the method that requires the lowest Neff to achieve 50% precision for the top *L* predicted contacts, requiring a Neff between 418 and 451. CCMPred (GREMLIN) required a Neff between 477 and 490 (480 and 492). These values are estimates only.

We then used the three contact predictors that achieved the highest precision (CCMPred, metaPSICOV stage 2 and GREMLIN) to assess the correlation between protein length and the precision of predicted contacts. Counter to what was previously reported (Kamisetty *et al.*, 2013), we found no correlation between the precision of contact predictions and protein length (Supplementary Fig. S6). We also normalized the number of sequences in the MSA according to protein length and found that this did not lead to any improvement in the correlation.

#### 3.1.1 Precision of predicted contacts and SCOP class

**Fig. 2:**
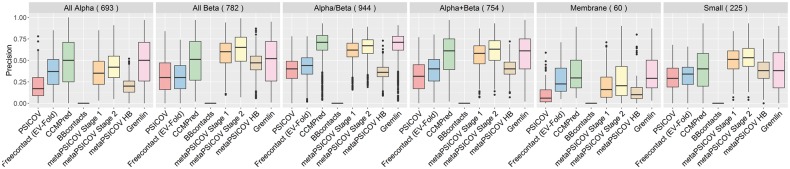
Precision of top *L* predicted contacts according to target’s SCOP class as output by eight contact predictors for 3458 proteins in our Astral dataset. Precision for the PDB-representative dataset including PConsC2 is shown in SI Figure S7

We assessed the precision of the predicted top *L* contacts within each of the six main SCOP classes (Fig. 2). MetaPSICOV Stage 2 had the highest average precision for 3 out of 6 SCOP classes, CCMPred/GREMLIN having the higher average precision for All-α and membrane proteins. The precision of all methods was generally bad for proteins belonging to the All-α and membrane SCOP class. Interestingly, metaPSICOV predictors performed worse for these two SCOP classes than for other classes. Generally, most methods performed well for α/β proteins and poorly for membrane proteins, but this is likely to be a reflection of the number of effective sequences in the MSAs according to SCOP class (Supplementary Fig. S8) in our dataset. Since Bbcontacts outputs a number of predictions smaller than L/5 for most cases, we also assessed the precision of the predicted top L/10 contacts within each SCOP class (Supplementary Fig. S9). For the top L/10 predictions, metaPSICOV stage 2 had the highest precision for 5 out of 6 SCOP classes and CCMPred had the highest average precision for membrane proteins.

**Fig. 3:**
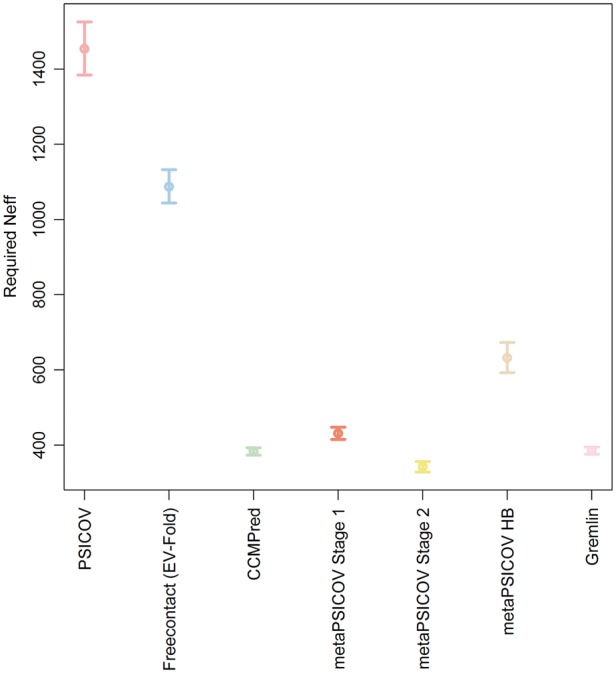
Confidence Intervals (95%) for the estimates of the number of effective sequences (Neff) required to produce contact predictions with a precision greater than 50% for seven contact predictors

To further investigate the cause of the lower precision of contacts predicted for All α proteins, we also assessed the precision of contacts predicted for different secondary structure types (Supplementary Fig. S10). This analysis was also meant to ascertain if there is a specific secondary structure type for which contact prediction is harder. Here, a contact predicted between two loop residues is described as loop-loop. Analogously, the remaining categories are loop–helix, loop–strand, helix–helix, helix–strand and strand–strand. When considering contacts formed between different secondary structure categories, metaPSICOV Stage 2 presented the highest average precision for four categories (loop–loop, loop–helix, loop–strand and helix–strand) and CCMPred/GREMLIN presented the highest average precision for helix–helix and strand–strand categories. Our results show that helix–helix contacts are not predicted significantly less well when compared to other secondary structure types, despite the fact that contacts predicted for All-α proteins tend to be less precise than for the other SCOP classes.

As observed for our Astral dataset, the true protein contacts are not evenly spread out across different secondary structure categories. It would be desirable to have the same proportion of predicted contacts per secondary structure type as observed for the true contacts. Therefore, we assessed the proportion of contacts predicted by each method for each secondary structure category (Supplementary Fig. S11). As expected, both bbcontacts and metaPSICOV-HB are enriched for predicted contacts formed by at least one β-strand residue. CCMPred and GREMLIN achieved the proportions that were the most similar to the true contact distribution for the secondary structure categories. MetaPSICOV stages 1 and 2 underpredict contacts formed between loop residues and overpredict strand-strand contacts. This lower prediction rate for the difficult to predict loop-loop contacts may be one of the reasons why the metapredictors are, on average, more precise.

### 3.2 Determining the best contact predictor to assist in template-free protein structure prediction

We have used the top *L* predicted contacts as output by seven of the eight contact predictors to assist in template-free protein structure prediction using the cotranslational predictor SAINT2. Predictions generated by GREMLIN were excluded from this analysis as they were redundant to predictions generated by the analogous method CCMPred.

For each contact predictor, we have used their predicted contacts with SAINT2 to generate 10 000 decoys for the 41 proteins in our PDB-Representative dataset. In total, 7×41×10 000=2 870 000 decoys were generated, using more than a decade of CPU-core hours. The TM-Score of the best decoy produced by SAINT2 assisted by predictions from each method and for each protein in our dataset are shown in Supplementary Figures S12 and S13, along with the results produced by SAINT2 without using any predicted contacts.

An initial assessment of the modelling results suggested that the best models produced by SAINT2 present similar qualities, regardless of the contact predictor used in model generation. Correct models (TM-Score > 0.5) were generated for 18 out of 41 cases and only two methods (CCMPred and metaPSICOV Stage 1) produced correct models for all of these 18 cases. SAINT2 without any contact information produced a correct model for six cases and produced the best model across all methods for only one case (Supplementary Fig. S20).

**Fig. 4:**
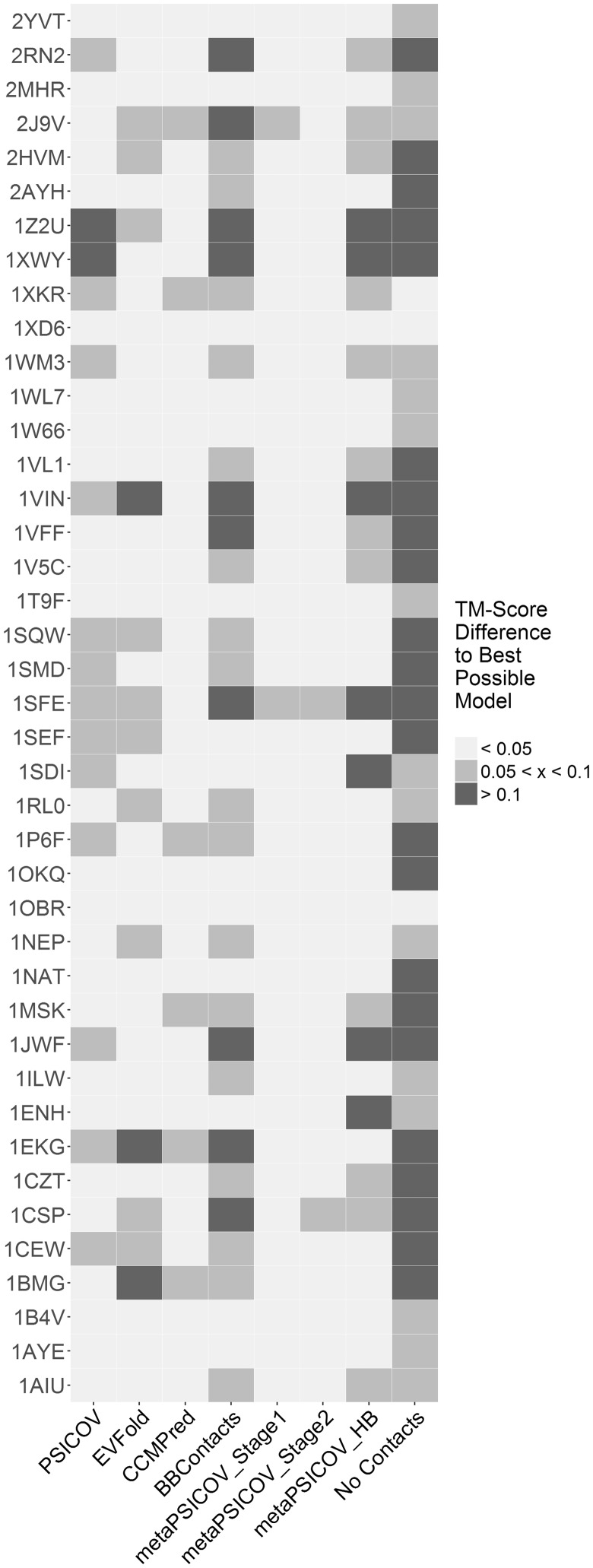
Difference in the TM-Score between the best possible model and the best model produced using each of seven contact predictors for the 41 proteins in our PDB-Representative dataset. We used SAINT2 with the predicted contacts output by each contact predictor to generate 10 000 decoys per target. Cases for which the best model produced using a given method is more than 0.1 (or more than 0.05, but less than 0.1) TM-Score units away from the best possible model are coloured in dark grey (light grey). Data is also shown for SAINT2 without using any predicted contact information (no contacts)

We assessed the difference in TM-Score between the best possible model against the best model produced by each of the contact predictors (Fig. 4). Our results show that metaPSICOV Stage 1 generated a model within 0.05 TM-Score units of the best possible model for 39 of the 41 cases.

When considering the SCOP Class of the targets (Supplementary Fig. S14), the best models produced with SAINT2 + metaPSICOV Stage 1 and SAINT2 + metaPSICOV Stage 2 were better than the ones generated by SAINT2 + any other contact predictors for α/β and α+β proteins. Comparing results for each SCOP class, metaPSICOV stage 1 produced, on average, better models for 3 out of 4 SCOP classes, with metaPSICOV stage 2 outperforming metaPSICOV stage 1 for All α proteins. Contrary to the precision of predicted contacts, SAINT2 produced better models for All-α proteins and worse models for All-β proteins compared to other SCOP classes. For All-α proteins, a small number of correct predicted contacts appears to be sufficient for accurate model generation. This may be due to α-helical fragments being inherently easier to model than β-strand fragments (de Oliveira *et al.*, 2015).

We assessed the influence of incorrect restraints on model quality by comparing the modelling results obtained using the most precise predictor (metaPSICOV stage 2, average precision = 66.5%) against the least precise predictor (PSICOV, average precision = 36.5%). This assessment was performed by comparing the precision of predicted contacts against the model quality produced by each approach for our PDB-representative dataset of 41 proteins (Supplementary Fig. S15). Our findings show a weak correlation between model quality and precision of predicted contacts, as previously reported in (Kosciolek and Jones, 2014). We also observed nine cases where a set of predictions with lower precision led to better models being produced. This suggests that the influence of incorrect restraints cannot be easily quantified.

### 3.3 Satisfied predicted contacts correlate with model quality

Predicted contacts have been shown to be useful in performing quality assessment of generated decoys (Kamisetty *et al.*, 2013). This is usually done by computing a score based on how many of the predicted contacts have been satisfied in a given model.

We have assessed whether satisfied predicted contacts, correct or otherwise, show good correlation with model quality. This comparison was performed in terms of short-range and long-range predicted contacts (Supplementary Fig. S16). For this analysis, we have selected only three contact predictors, CCMPred and metaPSICOV Stages 1 and 2, as their predictions led to the best models generated by SAINT2 in a majority of cases. Our results reveal that there is a correlation between model quality and the proportion of predicted contacts that are satisfied. Ranking decoys using the proportion of predicted satisfied contacts did not produce good results (Supplementary Fig. S22).

Given that the spread of predicted contacts has also been reported to be crucial for accurate topology modelling (Kim *et al.*, 2014), we investigated the correlation between model quality and the spread of satisfied long-range predicted contacts (Supplementary Fig. S17). Our results show no correlation between these two features, however there are only two cases in which the predicted contacts are not sufficiently evenly spread (one correctly predicted contact every 12 residues) (Kim *et al.*, 2014).

### 3.4 Detecting incorrect models using satisfied predicted contacts

We have assessed whether the proportion of satisfied predicted contacts can be used to discriminate between correct (TM-Score > 0.5) and incorrect models. For this analysis, we used the 10 000 decoys generated using SAINT2 and metaPSICOV stage 1 for each of the 41 protein in our PDB-representative dataset. We have considered the proportions of satisfied long-range, short-range and all-range contacts. Our results show that correct models tend to satisfy more of the predicted contacts than incorrect models (Supplementary Fig. S18). Separation was more pronounced for the satisfied predicted long-range contacts.

**Fig. 5:**
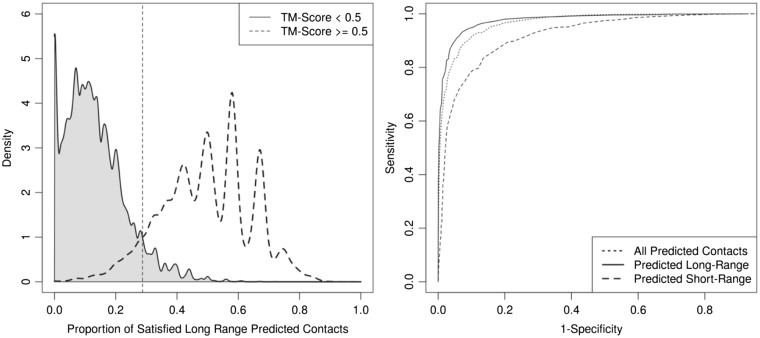
Naive classification of decoys as incorrect models amongst 10 000 decoys generated by SAINT2 and metaPSICOV stage 1 for the 41 proteins in our PDB-Representative dataset. The density estimates of the proportions of satisfied predicted long-range contacts for the two groups are shown on the left. The dashed line indicates the threshold (28.7%) that minimizes classification errors. The density estimates of the proportion of satisfied predicted short/all-range contacts is shown in SI Figure S18. To ascertain which measure led to the best classification of decoys, we varied the rejection threshold to build a ROC curve for each of the measures (right). Decoys that did not meet the varying cutoff of satisfied predicted short/long-all-range contacts were classified as incorrect. Predicted long-range contacts are shown to attain the best balance between sensitivity and specificity

We attempted to classify decoys as correct or incorrect based on a varying cutoff of satisfied predicted contacts. We built three naive classifiers based on the proportion of satisfied predicted long-range, short-range and all-range contacts. Using the optimal cutoff of 28.7% satisfied predicted long-range contacts to discard decoys, we obtain a false negative rate of 2478 out of 29 985 correct models while the proportion of correct decoys in the ensemble increases from 7.3% to 50.6%. Using the optimal cutoff for satisfied short-range (all-range) predicted contacts led to 4820 (2890) false negatives and increased the proportion of correct decoys to 30.6% (41.1%). Our results show that the proportion of satisfied long-range predicted contacts is the best indicator amongst the three ranking methods to perform model classification (Fig. 5).

It is desirable not to discard too many correct models for any particular case. Therefore, we assessed the impact of discarding decoys on the TM-Score Best and TM Score Top 5 (Supplementary Fig. S19). Our results show that, for the cases where a correct model was produced, we discarded the best decoy for only one case using our satisfied long-range predicted contact cutoff. When considering the TM-Score Top 5, that is the five decoys that SAINT2 scoring function would have ranked as the best, there is little difference before and after discarding decoys. This suggests that discarding decoys in this fashion is not detrimental to SAINT2’s final model quality. For the cases where a correct answer was identified by SAINT2’s scoring function, there was one case where discarding models classified as incorrect led to a significant improvement on the quality of the best decoy selected by SAINT2.

There were eight cases where all the decoys were discarded. For all eight cases, no correct model was produced by SAINT2, which suggests that the precision of satisfied predicted long-range contacts can be used in a case independent fashion to identify where modelling has failed (no correct models in the entire decoy set). This approach has an advantage over existing scoring-function methods since scores are usually not comparable across different test cases.

## 4 Discussion

We have compared eight different co-evolution contact prediction methods in terms of the precision of their top *L* predictions and shown that metaPSICOV Stage 2 and CCMPred/GREMLIN have the highest average precision amongst tested methods. When considering the top L/10, top L/5 and top L/2 predicted contacts, metaPSICOV Stage 2’s precision is higher than other methods. As expected, we observe a decrease in precision for all methods when more contact predictions are considered. We also find that metaPSICOV stage 2 achieves a precision greater than 50% for more cases. It is therefore the most consistent amongst the top 3 predictors. Our findings confirm previous results (Kosciolek and Jones, 2014; Monastyrskyy *et al.*, 2015) that metaPSICOV Stage 2 is, on average, the most precise contact predictor amongst a plethora of tested methods.

Given that long-range contacts have been reported to be more useful for *de novo* protein structure prediction, we have also assessed the precision of each contact predictor in terms of short-range and long-range contacts. MetaPSICOV Stage 1 has the highest precision for long-range predicted contacts and its long-range predicted contacts are overall more precise than its short-range predicted contacts. Yet, metaPSICOV Stage 1 outputs a smaller proportion of long-range contacts when compared to other methods such as metaPSICOV Stage 2 and CCMPred/GREMLIN. Our results also show that PSICOV tends to output more long-range predicted contacts than short-range ones and its proportion of long-range contacts was the highest amongst all methods.

In our analyses, we observed that all methods are generally worse at predicting contacts for proteins belonging to the All α and membrane SCOP class. Where the low precision for membrane proteins can be explained by a significantly lower Neff for that SCOP class, the average Neff for All α and All β protein in our set was comparable. The poor precision for All α proteins is also not explained by the precisions observed for predicted contacts between α-helical residues (helix-helix secondary structure type), which are comparable to other SS types.

We have used the contact predictions output by seven of the methods as input to SAINT2, a fragment-based *de novo* protein structure predictor. Our results show that metaPSICOV Stage 1 can be used to produce models that are consistently as good as the best models produced amongst all methods. Surprisingly the more precise predictions on the set (output by metaPSICOV stage 2) performed marginally worse than metaPSICOV stage 1 during the modelling stage. This suggests that there might be other factors at play during model generation other than precision, such as the precision of predicted long-range contacts and spread of predicted contacts.

In our pipeline, we have considered the top *L* predictions as restraints for SAINT2. We explored using only L/10 restraints, a more precise subset of predictions, and observed worse modelling results. Given the weak correlation between model quality and precision of predicted contacts/spread of correct contacts, the determination of a confidence threshold for the number of restraints to be included is non-trivial and deserves further investigation.

When considering the SCOP class of the targets, we failed to produce good models for the majority of All-β proteins using any set of predictions, despite the fact that contact predictions for this SCOP class had the highest precision. This reveals a limitation of SAINT2 concerning the accurate prediction of All-β protein structures, an issue that will need to be addressed in the future.

We have shown that the proportion of satisfied predicted contacts can be used effectively to classify models as correct or incorrect. A naive classification scheme using the proportion of satisfied long-range predicted contacts leads to the best results. One of the best models produced by SAINT2 was classified as incorrect by this approach. The classifiers built were simplistic, but we use them only to highlight a potential application of satisfied predicted contacts.

Despite the correlation shown between model quality and satisfied long-range predicted contacts, considering only long-range contacts during model generation led to poorer models being produced (Supplementary Fig. S21). We believe this to be because short-range contacts contain useful information about local structure that is not necessarily captured by the fragments in our fragment library.

We observed no improvement in SAINT2’s TM-Score Top 5 after models were discarded based on their proportion of satisfied predicted long-range contacts. SAINT2 uses a scoring function to rank decoys. Previous CASP iterations have shown that clustering methods tend to perform better at quality assessment than single-decoy methods such as SAINT2’s. For clustering-based approaches, enriching the decoy ensemble with correct models could lead to improvements. It should also lead to reductions in computational time as decoys marked as incorrect can be ignored in all subsequent steps.

Overall, our findings corroborate the finding that the use of predicted contacts can improve *de novo* model generation and that these predictions should be used whenever sufficient sequence information is available. However, we show that contact predictors with a significant difference in precision can be used to generate models of similar quality, which suggests more care should be given as to how these contacts predictions are used during the model generation steps. Finally, we show that contact predictions can be used to improve model quality assessment either by identifying cases where modelling has failed or by classifying between good and bad decoys, which are both steps forward towards the implementation of a qualitative decoy scoring scheme.

## Supplementary Material

Supplementary Data
