# Supplementary material for: Comparing co-evolution methods and their application to template-free protein structure prediction
Source: Bioinformatics. 2016 Sep 27;33(3):373–81. doi: 10.1093/bioinformatics/btw618 (PMC5860252; doi:10.1093/bioinformatics/btw618)
Supplement: Supplementary Data [file btw618_supp.zip › SI_Text.pdf]

Bioinformatics
doi.10.1093/bioinformatics/xxxxxx
Advance Access Publication Date: -
Original Paper

Structural Bioinformatics

Comparing co-evolution methods and their application to template-free protein structure prediction.

Saulo Henrique Pires de Oliveira [1,*], Jiye Shi [2,3] and Charlotte M. Deane [1]

Abstract

Supplementary Material

© The Author 2016. Published by Oxford University Press. All rights reserved. For permissions, please e-mail: journals.permissions@oup.com

1 Supplementary Text 1: SAINT2

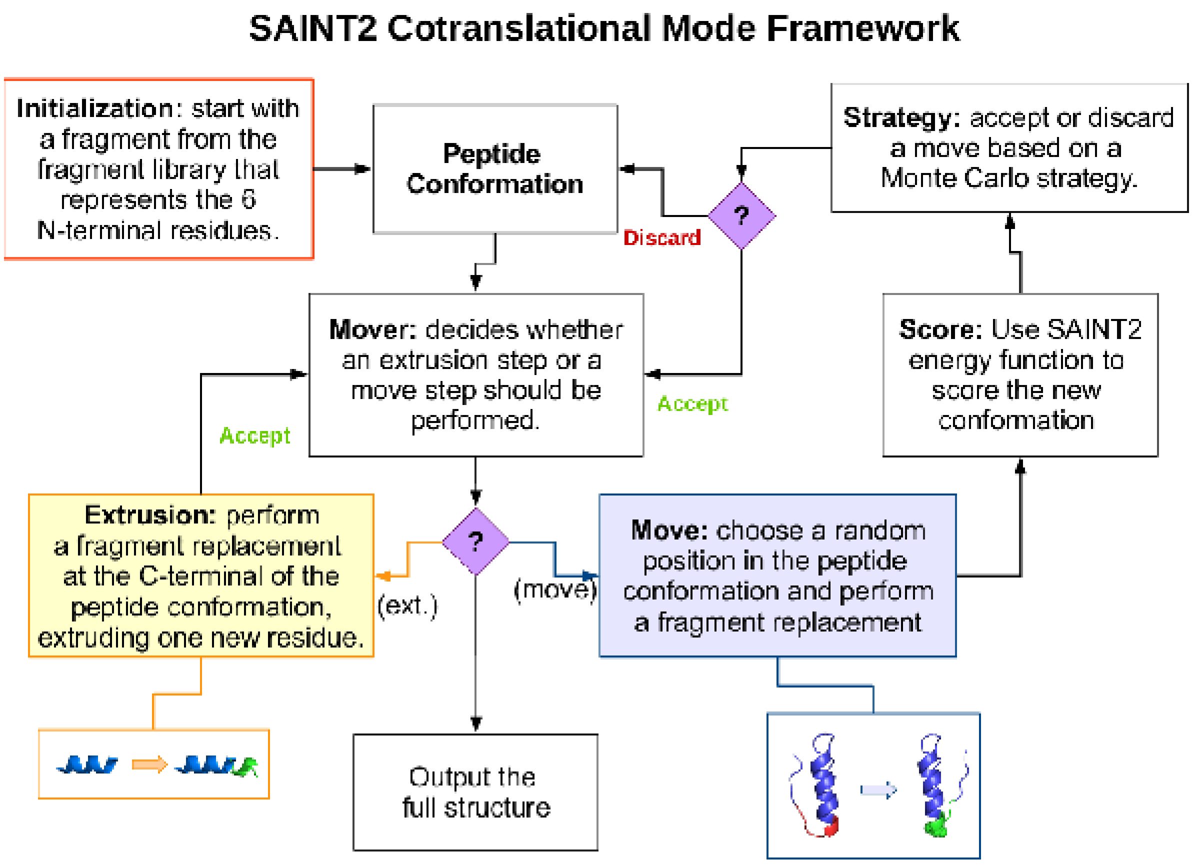

Fig. 1. The framework of the Cotranslational mode of SAINT2. The process is initiated by the selection of fragment from the library for the conformation of the six N-terminal residues. The Mover then decides whether moves or extrusions should be performed. In an extrusion, the peptide chain is extended by adding a fragment from the library to the C-terminus of the model. In a move, a fragment replacement is performed at a random position in the peptide. The resulting conformation is scored and either accepted or discarded according to a Monte Carlo strategy. Once the peptide has been fully extruded and the required number of moves has been performed, the full peptide conformation is output. This process generates a single decoy and, in practice, it is repeated thousands of times to generate a population of decoys.

SAINT2 is a fragment-assembly *de novo* structure prediction method. SAINT2 samples the conformational space using a library of fragments of known structure (SI Figure 1). Its scoring function is a combination of several different knowledge-based potentials.

1.1 SAINT2: Contact Potential

SAINT2 contains a predicted contact potential in its scoring function. The input of SAINT2's Contact Potential is a list of predicted contact pairs and their coupling score. There is no limit as to how many predicted contacts can be used as input to SAINT2. A score penalty is assigned to each predicted contact that is not satisfied in a given conformation. The penalty is linearly proportional to the distance between the pair of residues that were predicted to be in contact. The score of a given pair of residues i, j predicted to be in contact is given by:

$$S_{ij}^{contact} = \begin{cases} 0, \text{ if } ||\mathbf{C}_\beta(i) - \mathbf{C}_\beta(j)|| < 8.0 \text{ Å} \\ ||\mathbf{C}_\beta(i) - \mathbf{C}_\beta(j)|| - 8.0 \text{ Å}, \text{ otherwise.} \end{cases}$$

Where $\mathbf{C}_\beta(i)$ and $\mathbf{C}_\beta(j)$ represent the coordinates of the C-βs (C-αs in the case of glycine) of residues i and j and:

$$||\mathbf{C}_\beta(i) - \mathbf{C}_\beta(j)|| = \sqrt{\sum_{\kappa=x,y,z} (C_\beta^\kappa(i) - C_\beta^\kappa(j))^2}$$

The predicted contact score of a given conformation is the sum of the scores of every pair of residues predicted to be in contact. The time complexity of the predicted contact score is $O(L_{cont})$, where L_{cont} is the number of predicted contacts used as input (usually, $L_{cont} = L$).

SAINT2 uses contact information by adding a linear contact penalty score. Other contact penalty scores have been explored in SAINT2, for example those described in (1) and the ones described in (2). We compared the performance of SAINT2 using each of the three scores. The linear penalty described above was shown to produce marginally better results for a training set of 43 structurally diverse proteins. It may be the case that our training set did not contain a sufficient number of cases where contact predictions with poor precision were output. It is unclear which score would perform better for cases where a majority of predictions is incorrect, since linear penalty scores have been shown not to be robust against incorrect contact predictions.

2 Supplementary Text 2: SAINT2 Scoring Function

SAINT2 uses a knowledge-based potential built from the following components:

- RAPDF potential (3).
- Lennard-Jones potential (4).
- Solvation potential, as implemented in (5).
- Predicted secondary structure potential.
- Predicted torsion angle potential.
- Predicted inter-residue contacts potential (see SI Text 1).
- Orientation potential, as implemented in (5).

SAINT2's score is a weighted sum of each of its components. The score components are normalized by chain length and to a similar scale in order to ensure that the scoring weights reflect the contribution of each individual potential. This assists in comparing and understanding the contribution of each individual component to the final score.

References

[1]Kosciolek, T., and Jones, D.T. (2014) De novo structure prediction of globular proteins aided by sequence variation-derived contacts. *PloS one*, 9(3):e92197, 2014.

[2]Ovchinnikov, S., Kinch, L., Park, H., Liao, Y., Pei, J., Kim, D. E., Kamisetty, H., Grishin, N. V., and Baker, D. (2015). Large-scale determination of previously unsolved protein structures using evolutionary information. *Elife*, 4:e09248, 2015.

[3]Samudrala, R. and Moult, J. (1998). An all-atom distance-dependent conditional probability discriminatory function for protein structure prediction. *Journal of molecular biology*, 275(5):895–916.

[4]Jones, J. E. (1924). On the determination of molecular fields. ii. from the equation of state of a gas. In *Proceedings of the Royal Society of London A: Mathematical, Physical and Engineering Sciences*, volume 106, pages 463–477. The Royal Society.

[5]Tosatto, S. C. (2005). The victor/frst function for model quality estimation. *Journal of Computational Biology*, 12(10):1316–1327.
