# Supplementary material for: Comparing co-evolution methods and their application to template-free protein structure prediction
Source: Bioinformatics. 2016 Sep 27;33(3):373–81. doi: 10.1093/bioinformatics/btw618 (PMC5860252; doi:10.1093/bioinformatics/btw618)
Supplement: Supplementary Data [file btw618_supp.zip › SI_Table1.pdf]

Bioinformatics
doi.10.1093/bioinformatics/xxxxxx
Advance Access Publication Date: -
Original Paper

Structural Bioinformatics

Comparing co-evolution methods and their application to template-free protein structure prediction.

Saulo Henrique Pires de Oliveira [1,*], Jiye Shi [2,3] and Charlotte M. Deane [1]

Abstract

Supplementary Table 1

© The Author 2016. Published by Oxford University Press. All rights reserved. For permissions, please e-mail: journals.permissions@oup.com

Table 1. The 41 proteins comprising our PDB-Representative data set separated by SCOP classes. Proteins are single-domain, single chain, and belong to distinct PFam families.

**PDB ID**	**Pfam Family ID**	**Protein Length**	**Resolution**
SCOP Class α/β			
1AIU	PF00085	105 Residues	2.00 Å
1NAT	PF00072	124 Residues	2.45 Å
2RN2	PF00075	155 Residues	1.48 Å
1ILW	PF00857	180 Residues	2.05 Å
1VL1	PF01182	232 Residues	1.55 Å
1XWY	PF01026	264 Residues	2.00 Å
2HVM	PF00704	273 Residues	1.80 Å
1OBR	PF00246	326 Residues	2.3 Å
1VFF	PF00232	423 Residues	2.50 Å
1SMD	PF00128	496 Residues	1.6 Å
SCOP Class $\alpha + \beta$			
1WM3	PF00240	72 Residues	1.2 Å
1CEW	PF00031	107 Residues	2.0 Å
1EKG	PF01491	127 Residues	1.8 Å
1Z2U	PF00179	150 Residues	1.1 Å
1SQW	PF03657	188 Residues	1.90 Å
1XKR	PF04509	203 Residues	1.75 Å
1W66	PF03099	232 Residues	1.08 Å
1RL0	PF00161	255 Residues	1.4 Å
2YVT	PF12850	260 Residues	1.60 Å
1MSK	PF02965	331 Residues	1.8 Å
1AYE	PF02244	401 Residues	1.8 Å
1B4V	PF00732	504 Residues	1.5 Å
SCOP Class All β			
1CSP	PF00313	67 Residues	2.45 Å
1BMG	PF13895	98 Residues	2.5 Å
1XD6	PF01453	112 Residues	2.0 Å
1NEP	PF02221	130 Residues	1.7 Å
1CZT	PF00754	160 Residues	1.87 Å
1T9F	PF02815	187 Residues	2.00 Å
2AYH	PF00722	214 Residues	1.6 Å
1P6F	PF13895	241 Residues	2.2 Å
1SEF	PF07883	274 Residues	2.05 Å
1WL7	PF04616	312 Residues	1.9 Å
1OKQ	PF00054	394 Residues	2.80 Å
SCOP Class All α			
1ENH	PF00046	54 Residues	2.1 Å
2J9V	PF03997	99 Residues	2.0 Å
2MHR	PF01814	118 Residues	1.3 Å
1JWF	PF00790	147 Residues	2.10 Å
1SFE	PF01035	180 Residues	2.10 Å
1SDI	PF04356	213 Residues	1.65 Å
1VIN	PF00134	268 Residues	2.0 Å
1V5C	PF01270	386 Residues	2.0 Å

1 Supplementary Table 1

We used a set of 41 structurally diverse proteins extracted from the PDB. These proteins were selected to comprise a fair representation of the structures of the PDB. They are single chain, single domain proteins proportionally distributed into the four SCOP protein classes: all α, all β, α/β, and $\alpha + \beta$. They are also evenly spread in terms of length, ranging from 54 to 504 residues.
