# Supplementary figures and images for: Comparing co-evolution methods and their application to template-free protein structure prediction
Source: Bioinformatics. 2016 Sep 27;33(3):373–81. doi: 10.1093/bioinformatics/btw618 (PMC5860252; doi:10.1093/bioinformatics/btw618)

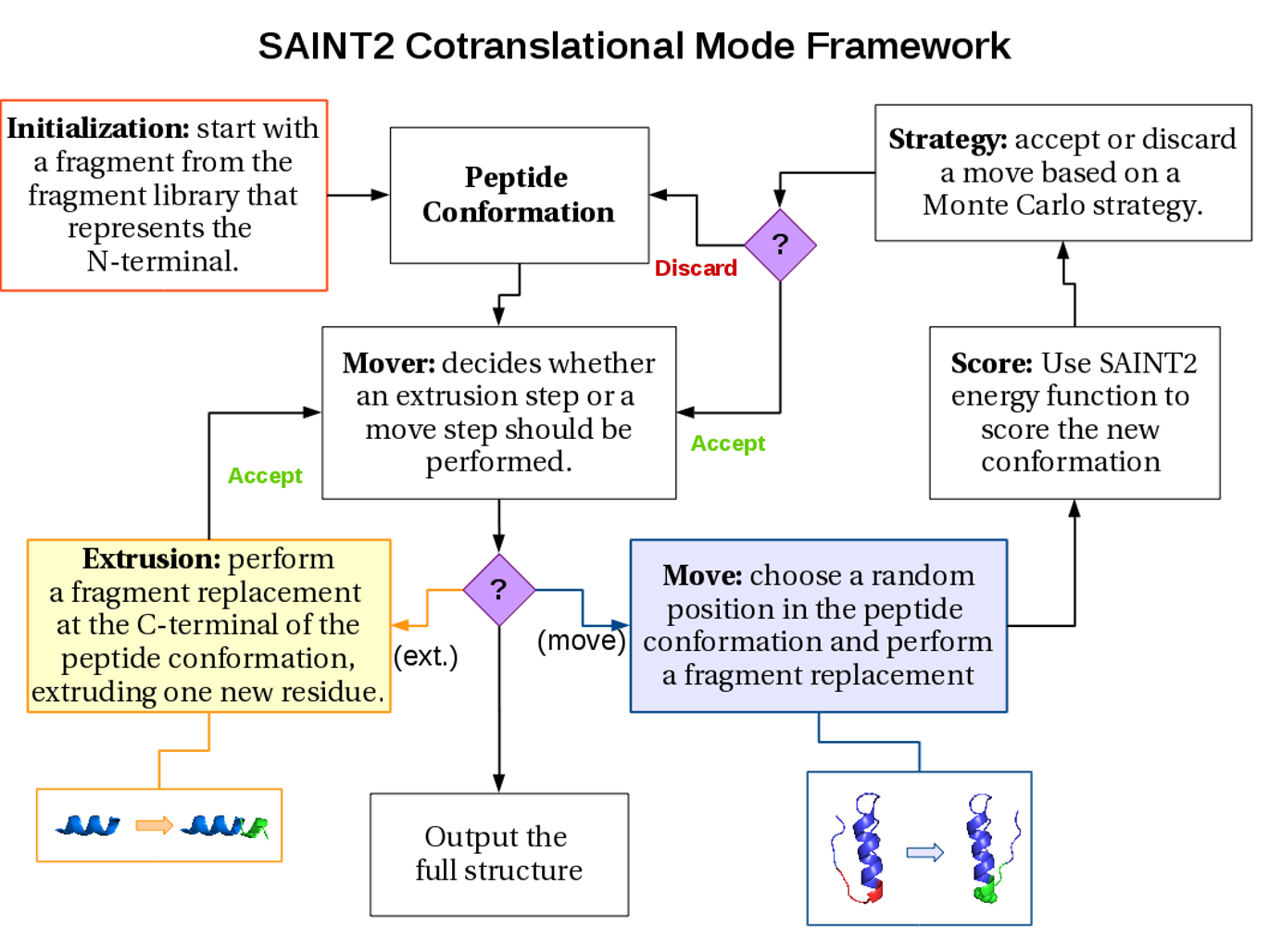

Supplement: Supplementary Data [file btw618_supp.zip › SI_Figure1.png]

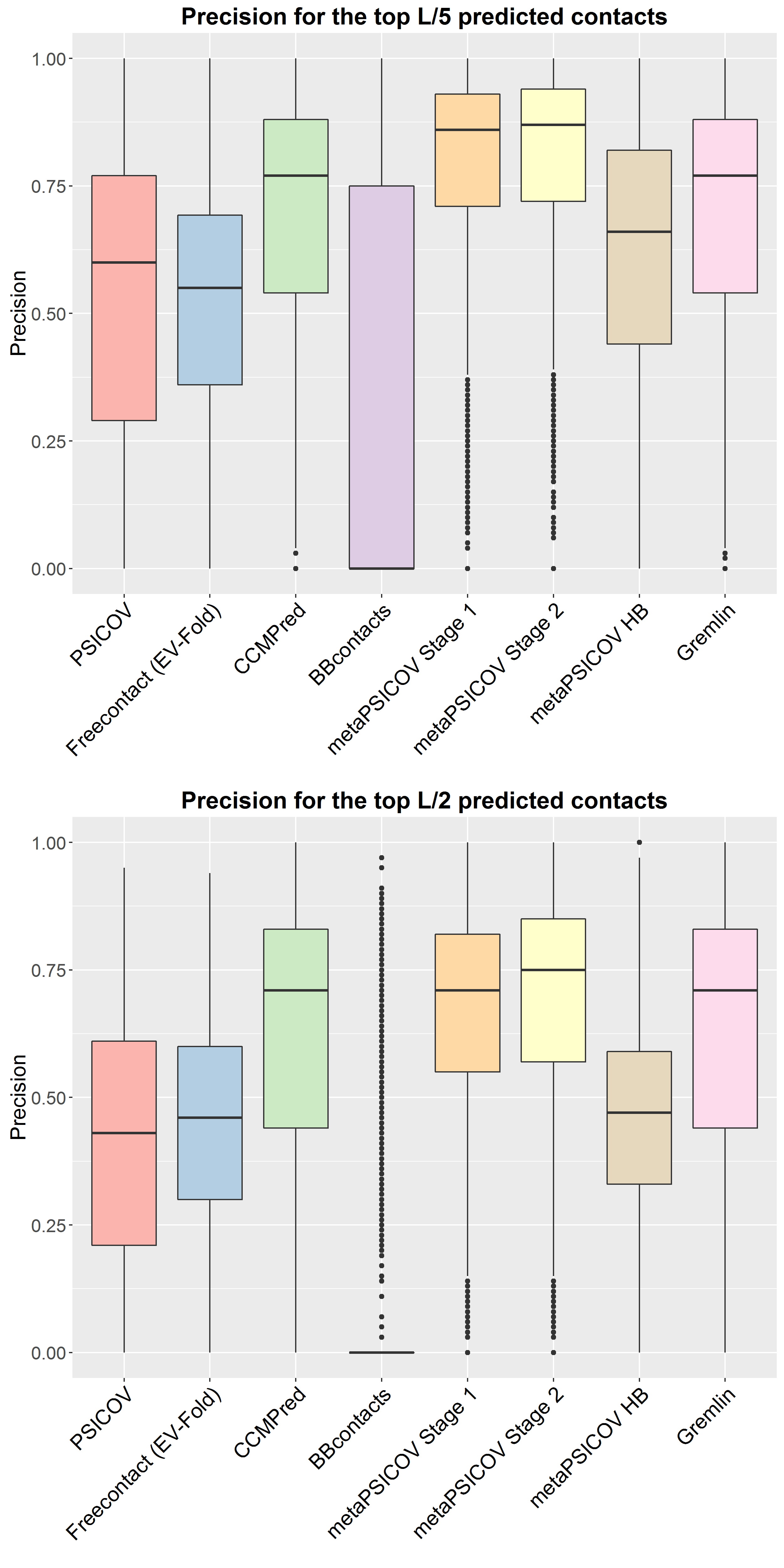

Supplement: Supplementary Data [file btw618_supp.zip › SI_Figure2.png]

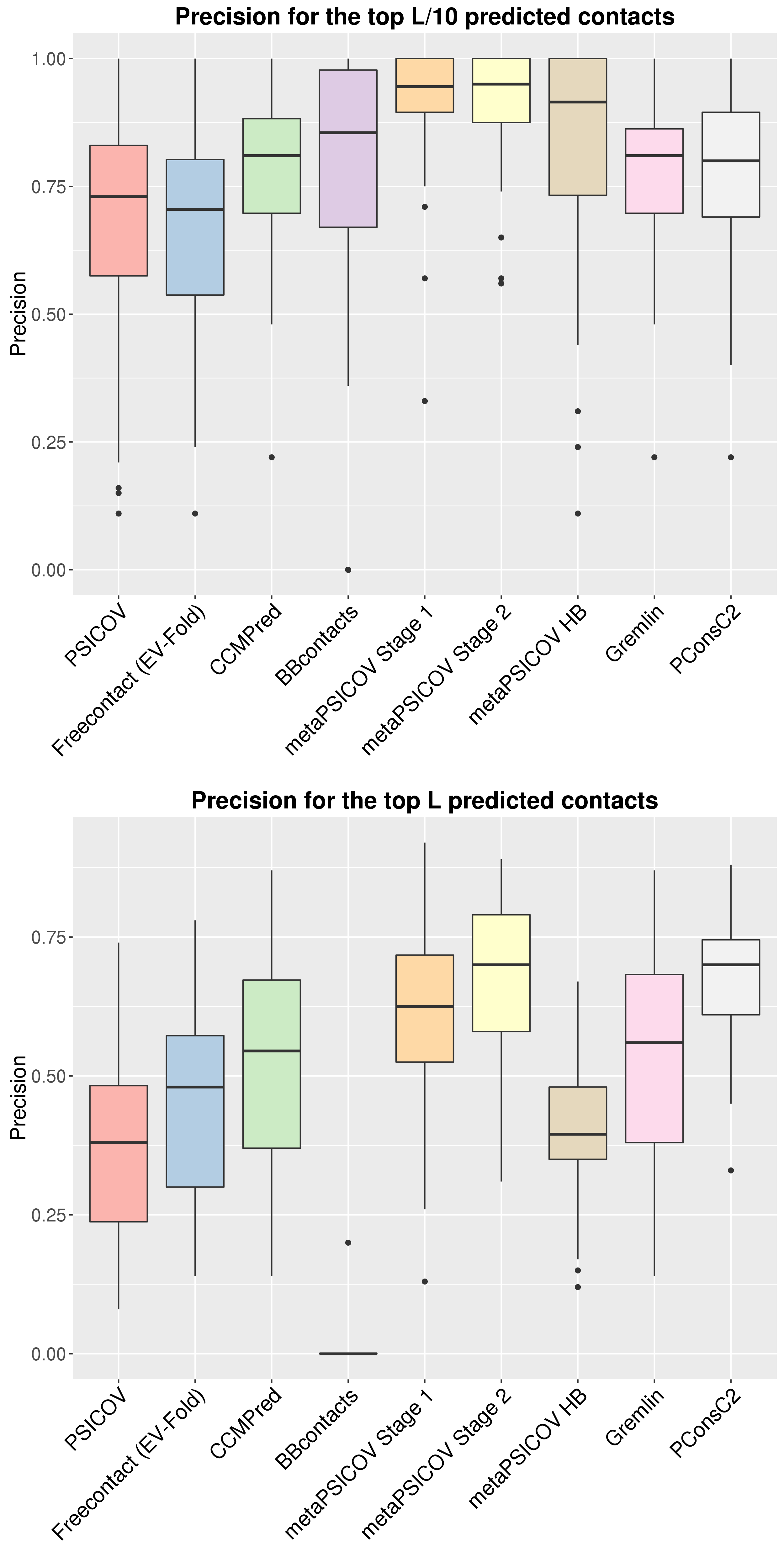

Supplement: Supplementary Data [file btw618_supp.zip › SI_Figure3.png]

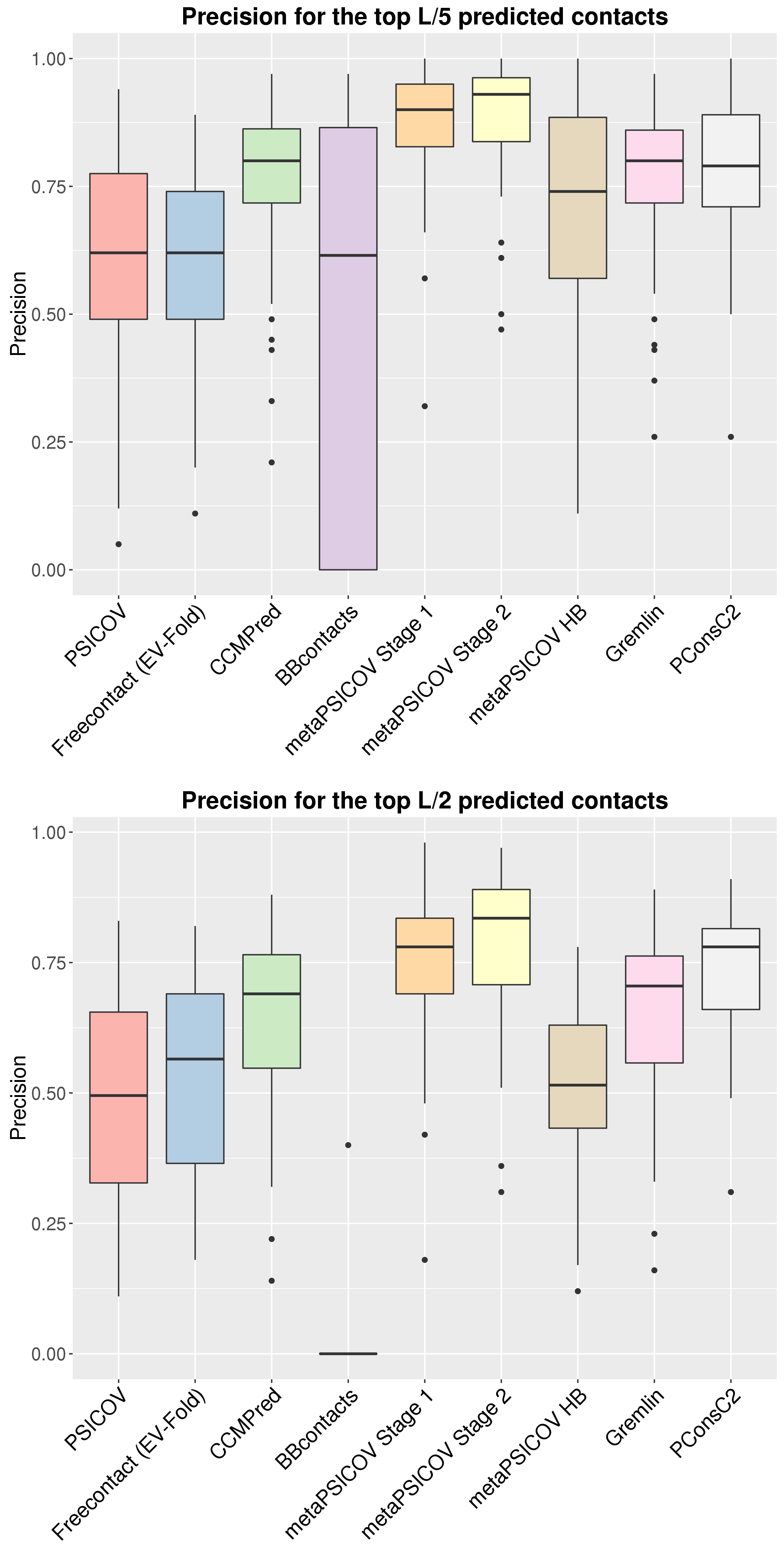

Supplement: Supplementary Data [file btw618_supp.zip › SI_Figure4.png]

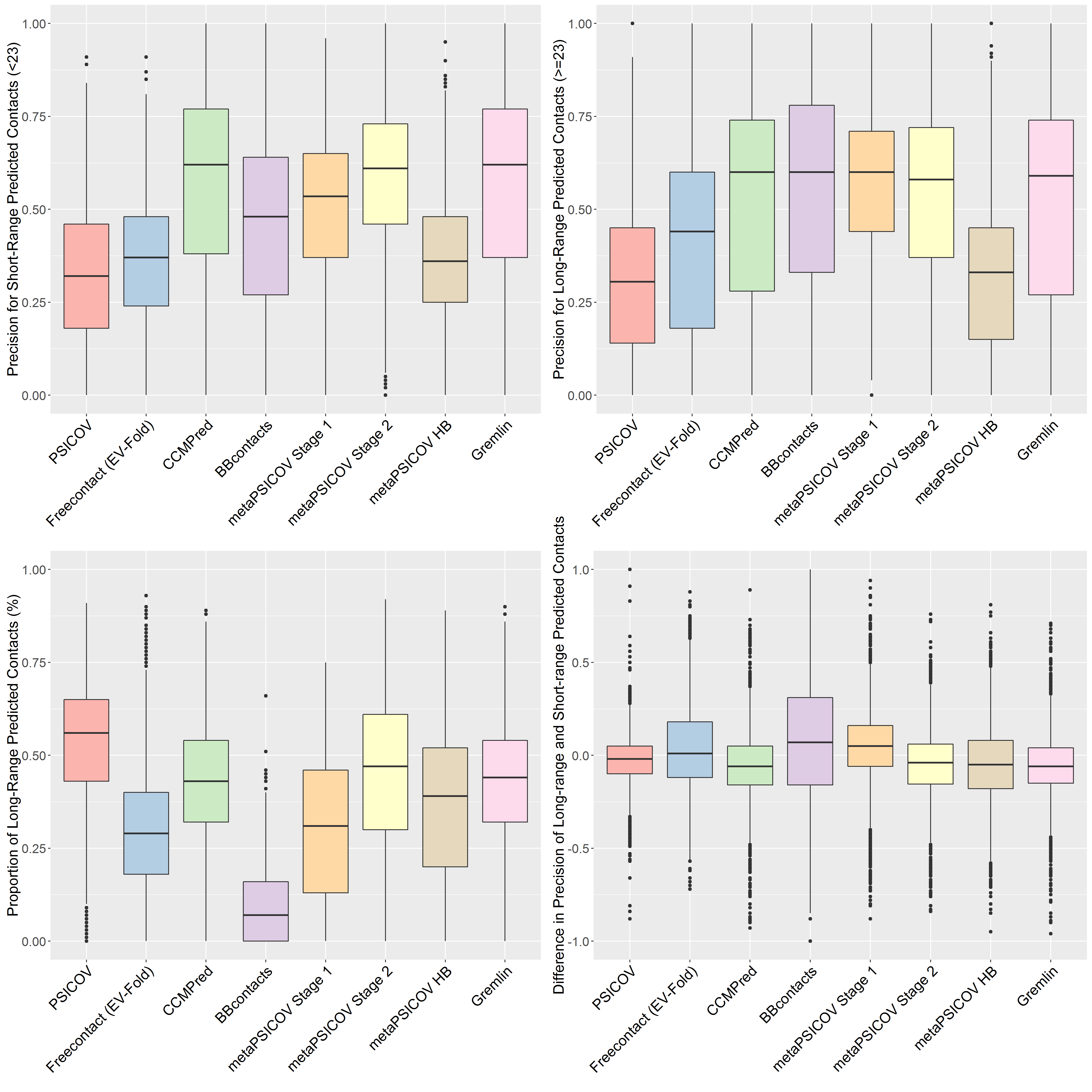

Supplement: Supplementary Data [file btw618_supp.zip › SI_Figure5.png]

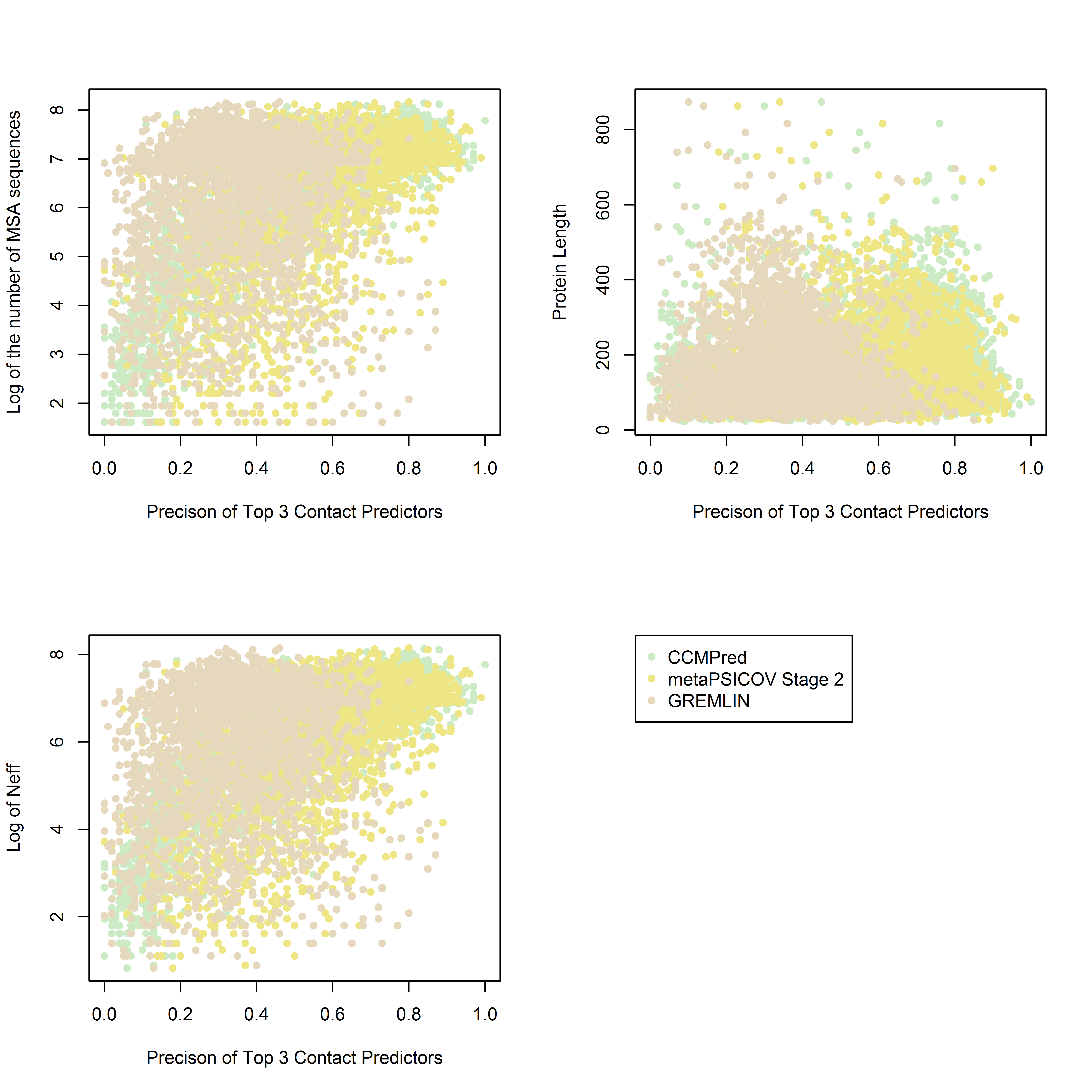

Supplement: Supplementary Data [file btw618_supp.zip › SI_Figure6.png]

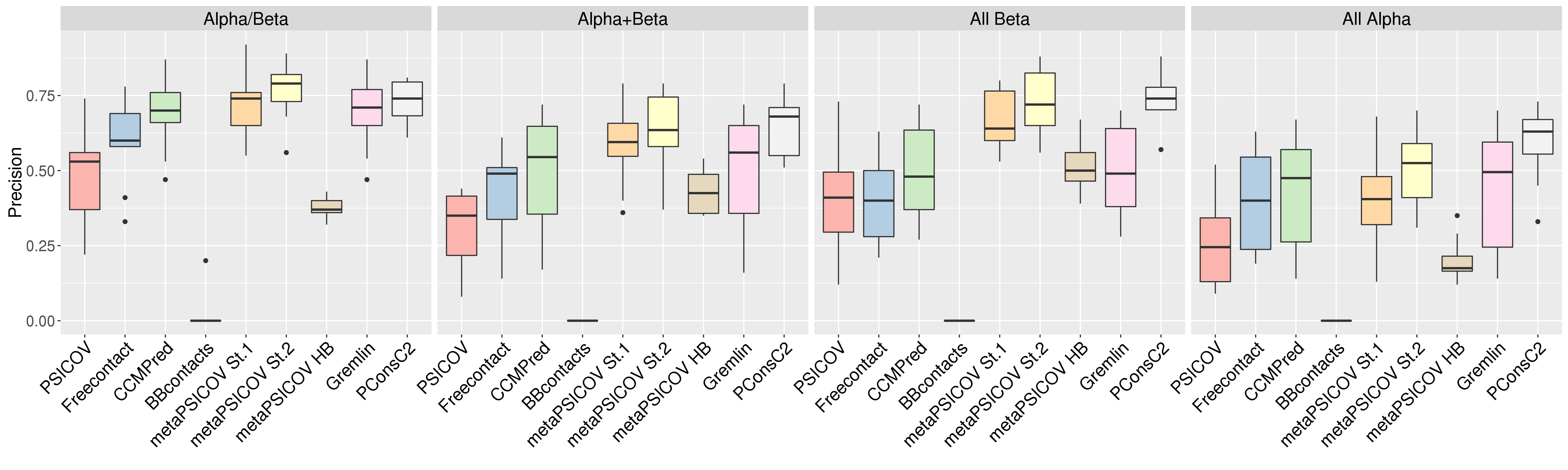

Supplement: Supplementary Data [file btw618_supp.zip › SI_Figure7.png]

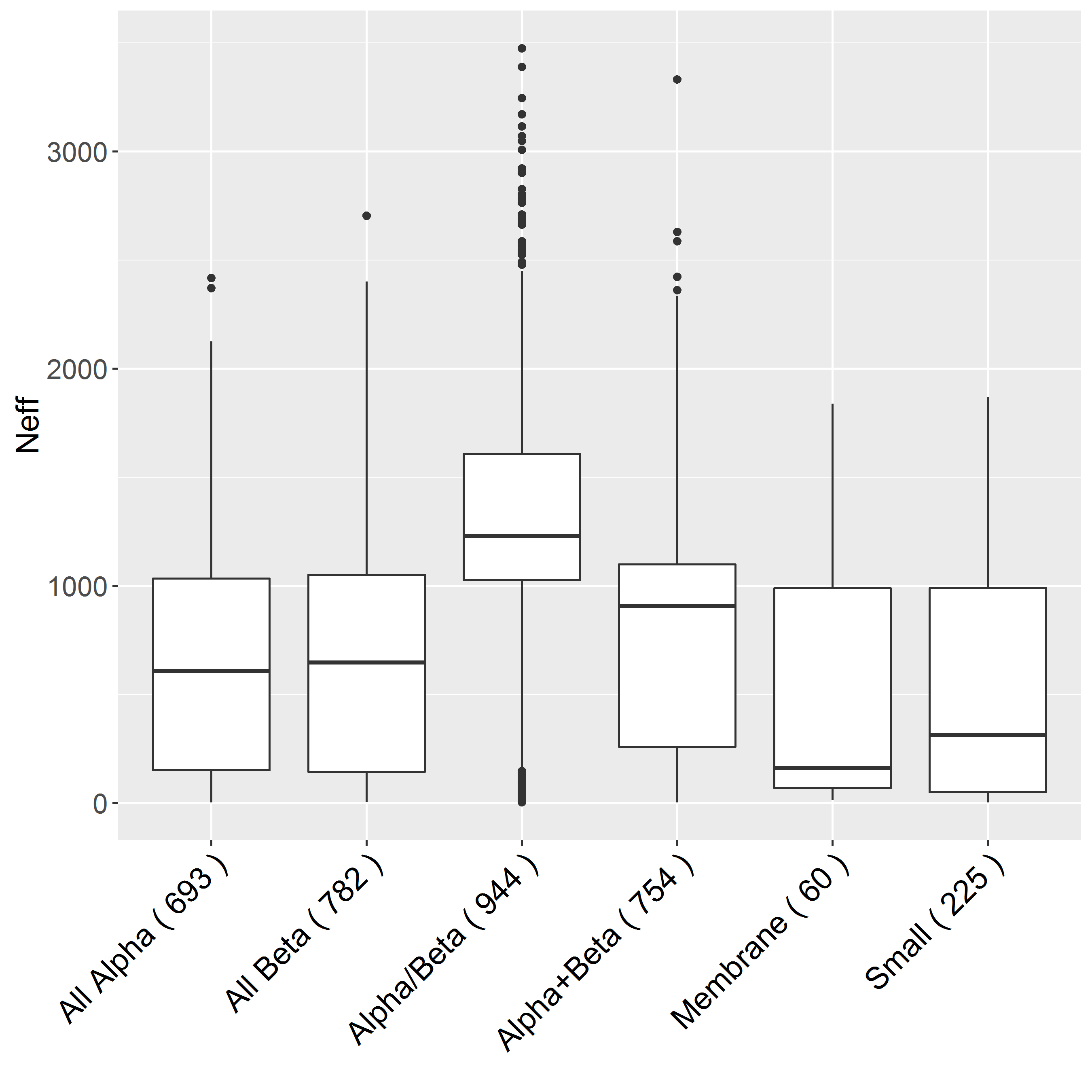

Supplement: Supplementary Data [file btw618_supp.zip › SI_Figure8.png]

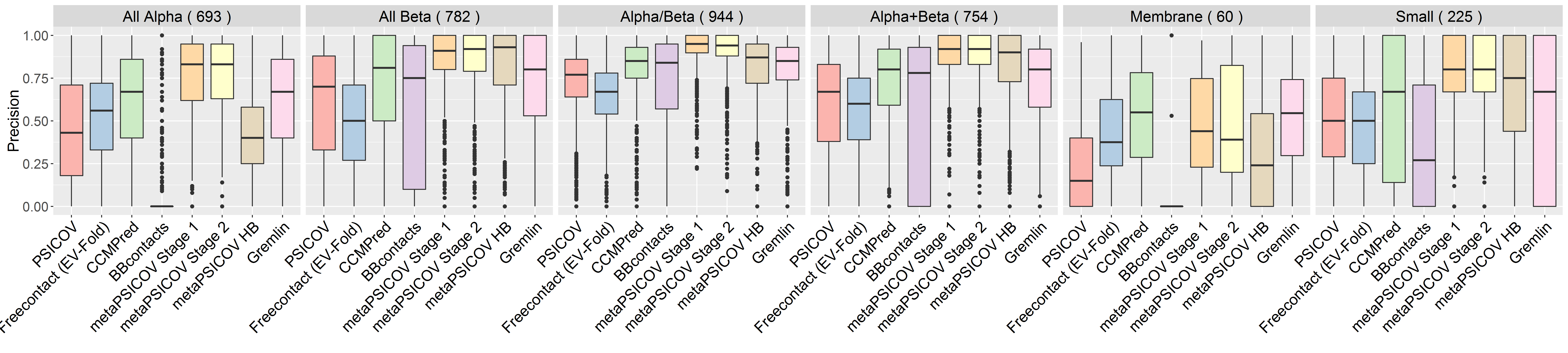

Supplement: Supplementary Data [file btw618_supp.zip › SI_Figure9.png]

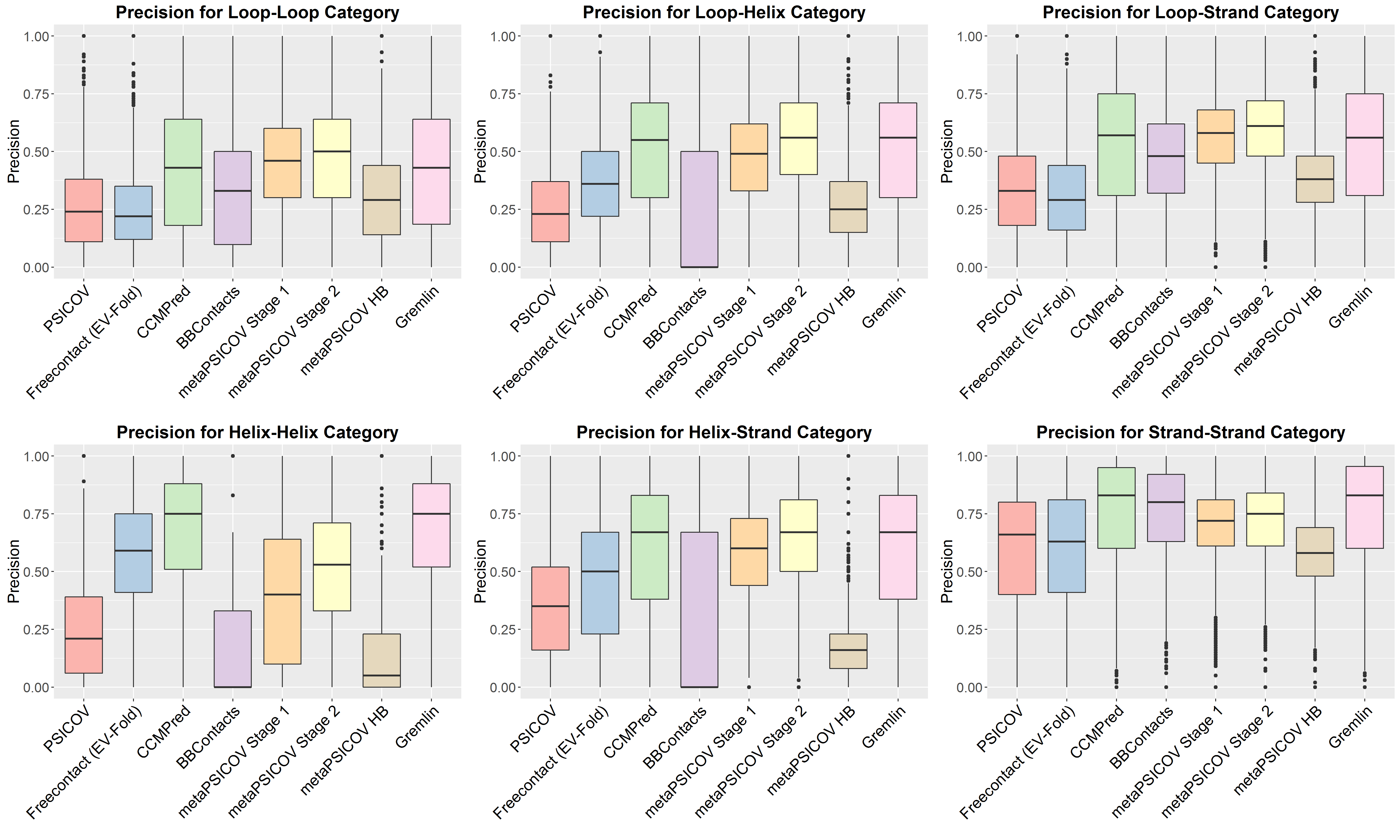

Supplement: Supplementary Data [file btw618_supp.zip › SI_Figure10.png]

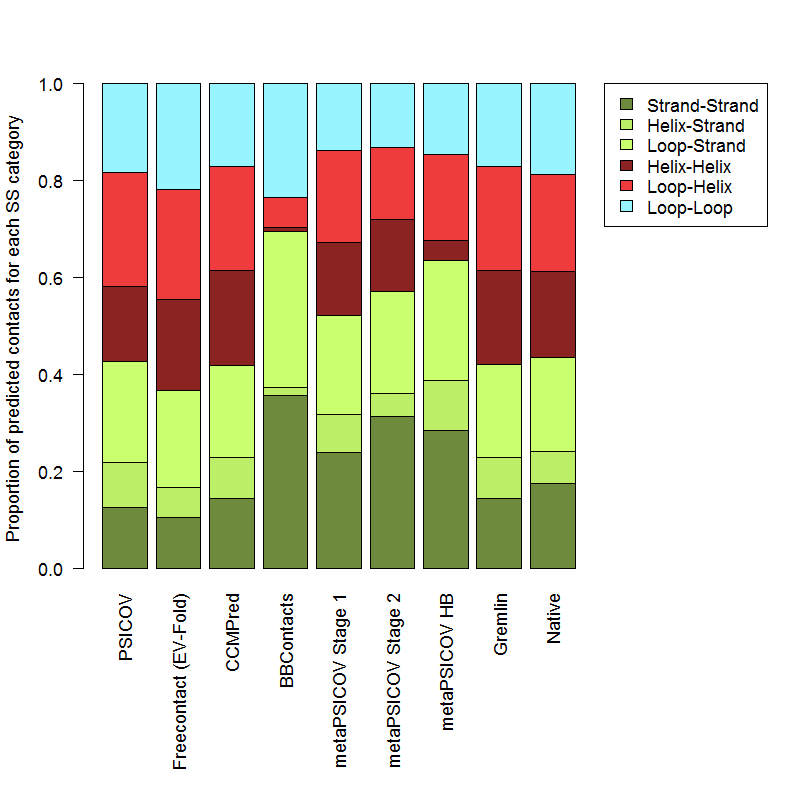

Supplement: Supplementary Data [file btw618_supp.zip › SI_Figure11.png]

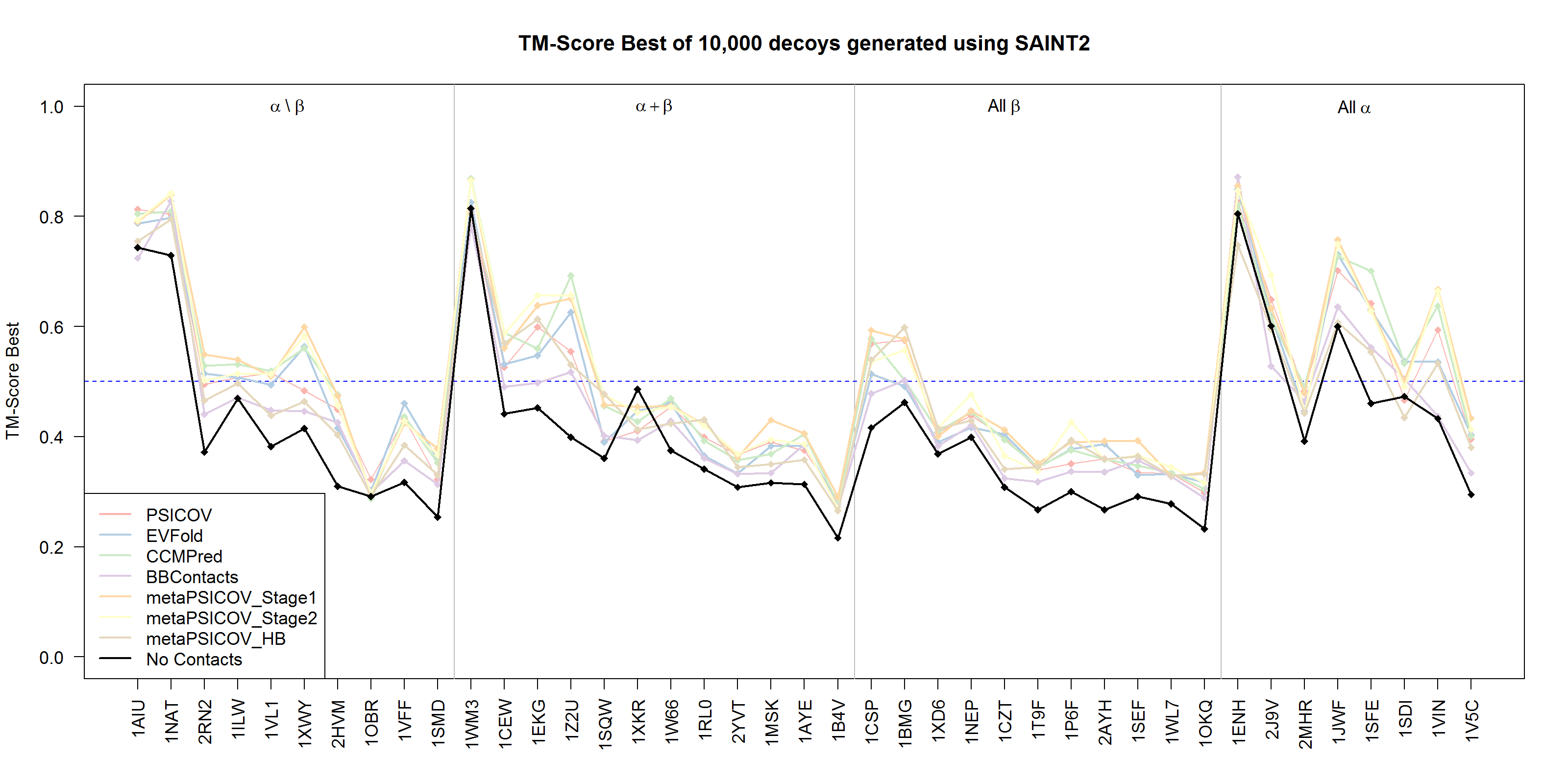

Supplement: Supplementary Data [file btw618_supp.zip › SI_Figure12.png]

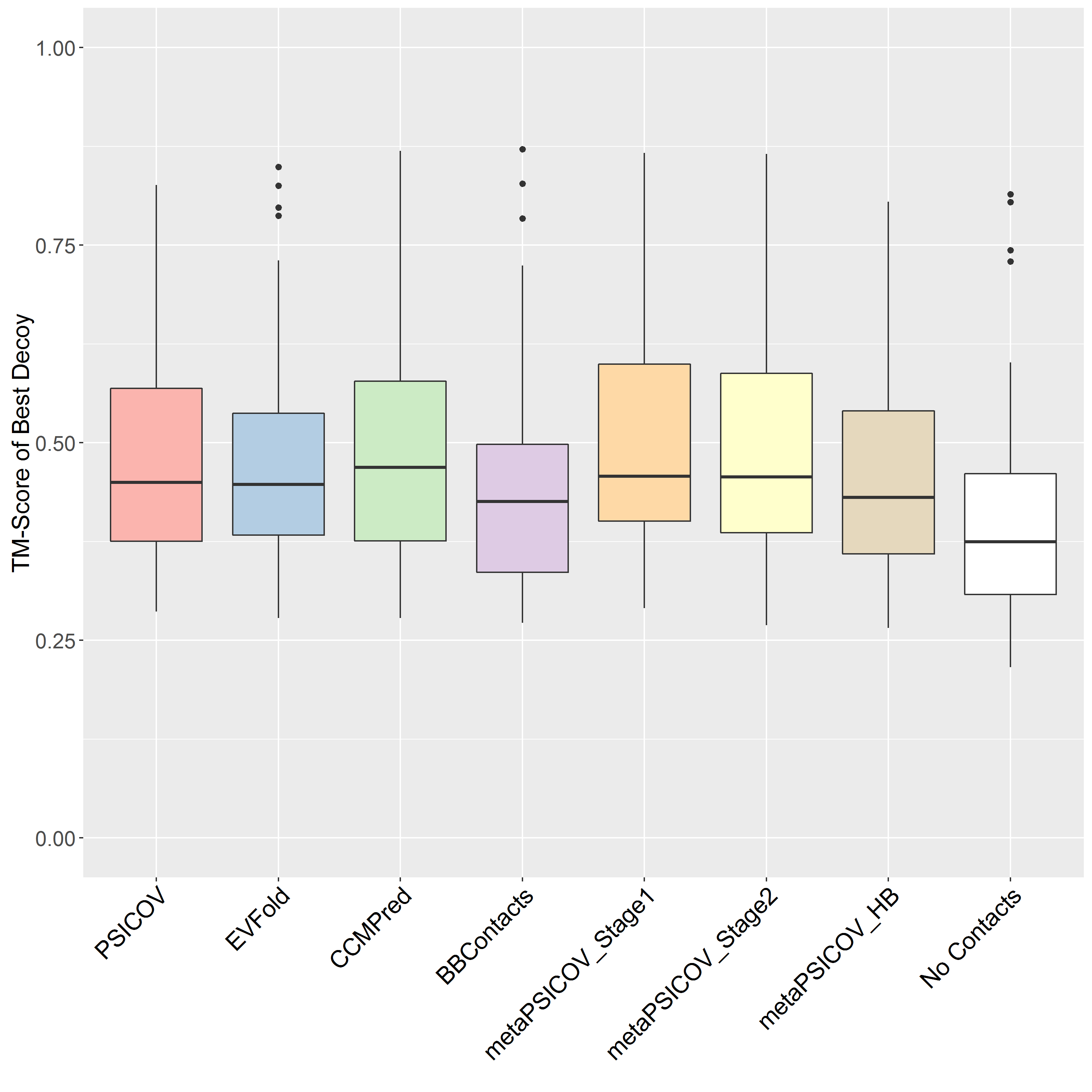

Supplement: Supplementary Data [file btw618_supp.zip › SI_Figure13.png]

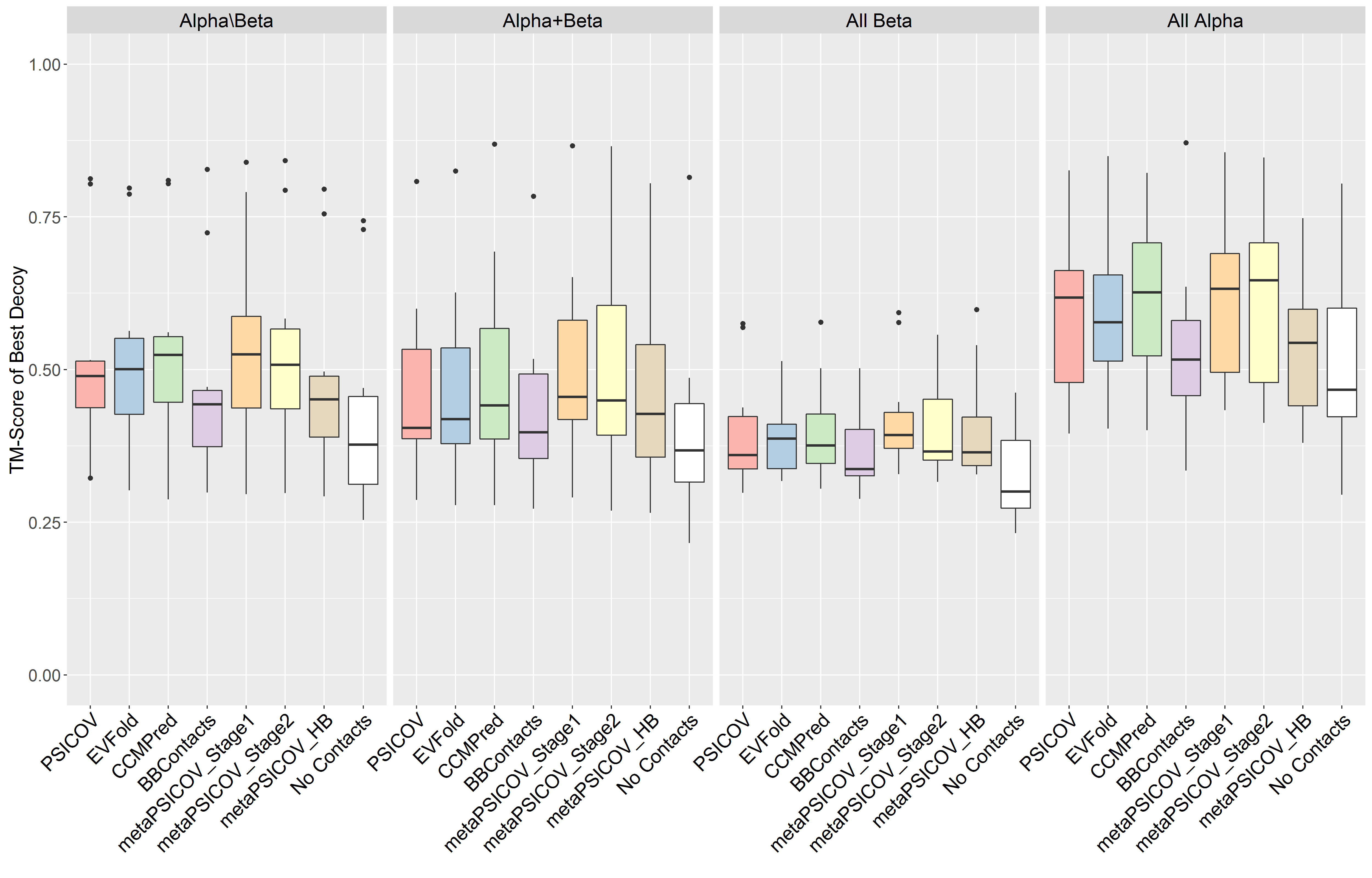

Supplement: Supplementary Data [file btw618_supp.zip › SI_Figure14.png]

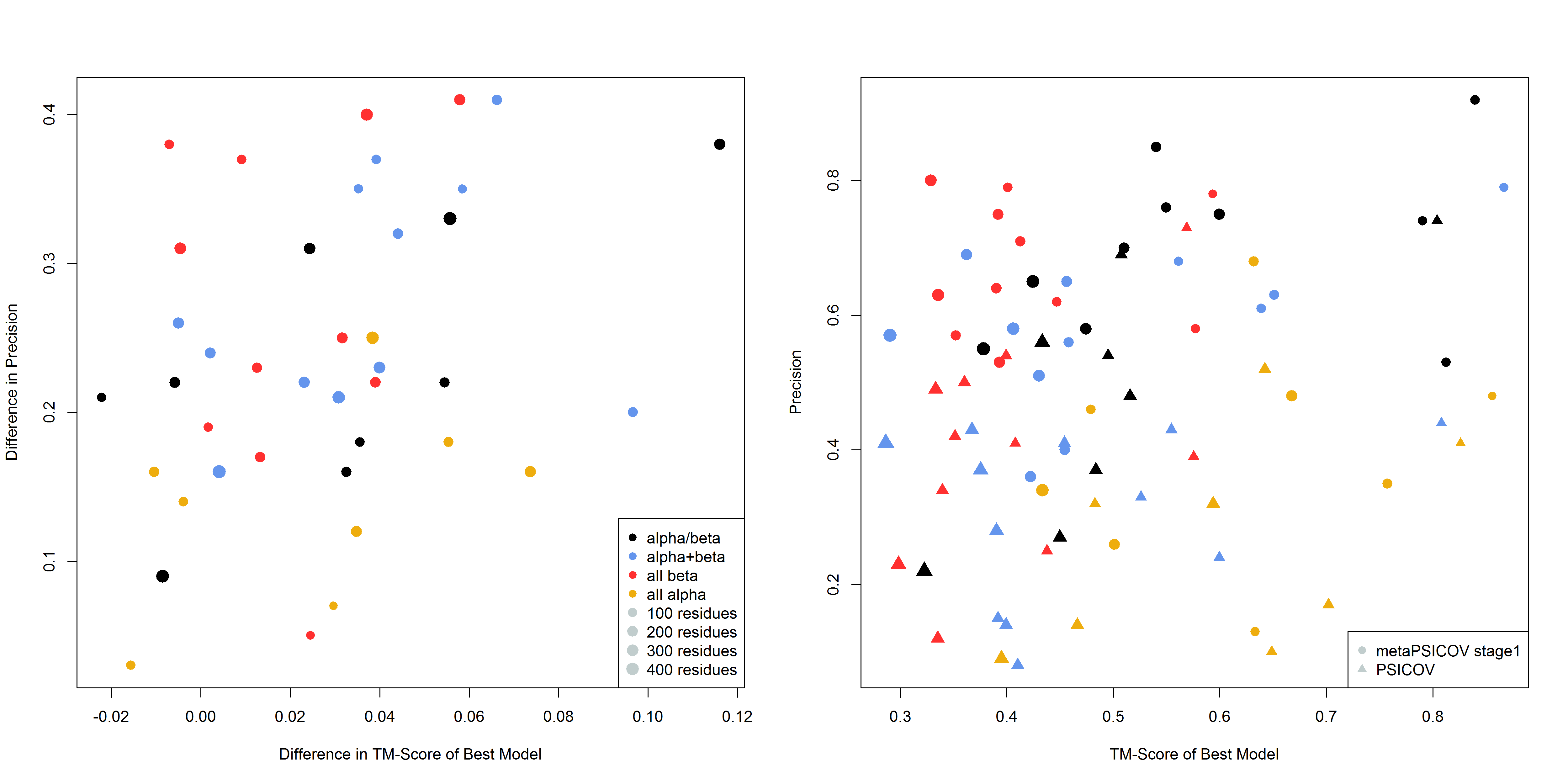

Supplement: Supplementary Data [file btw618_supp.zip › SI_Figure15.png]

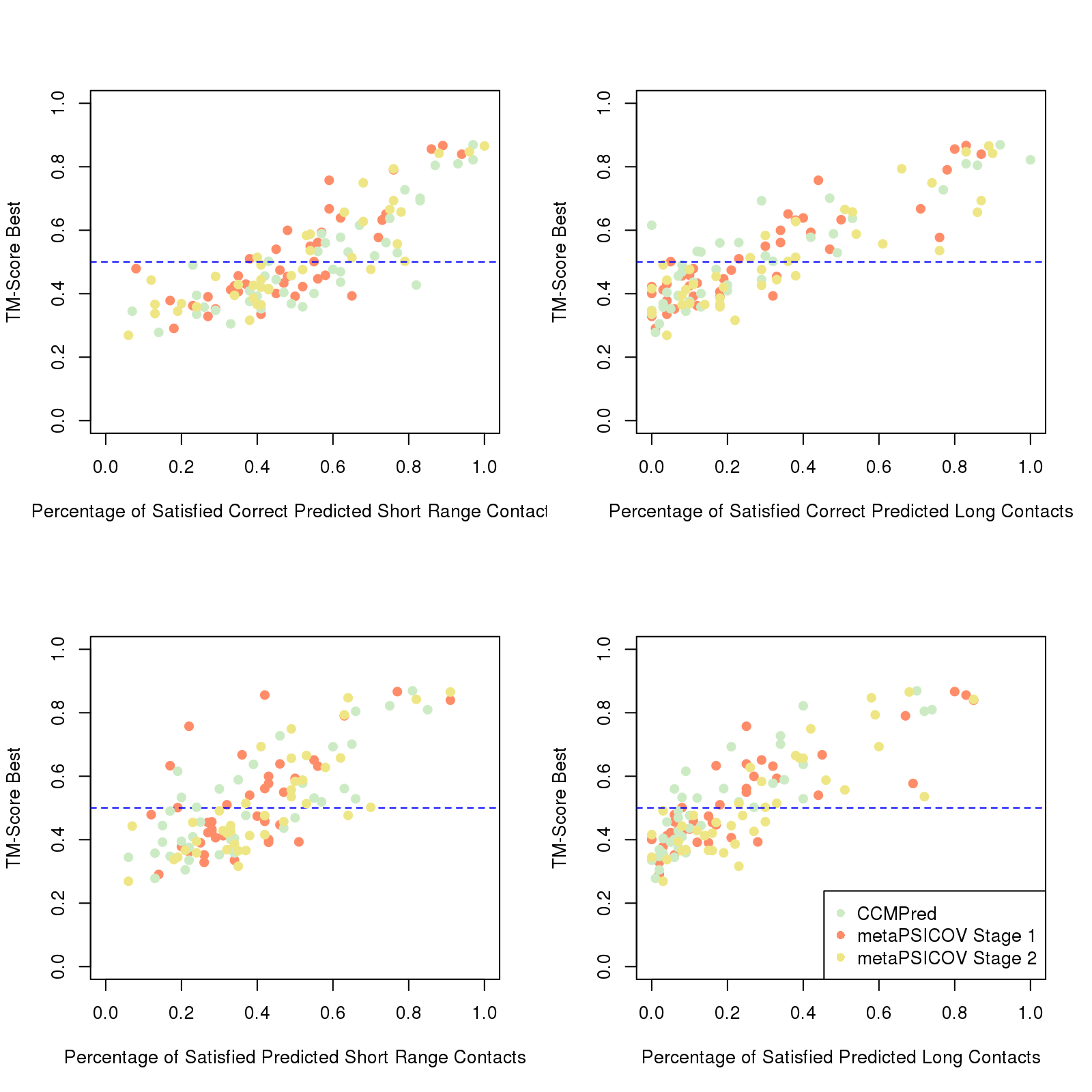

Supplement: Supplementary Data [file btw618_supp.zip › SI_Figure16.png]

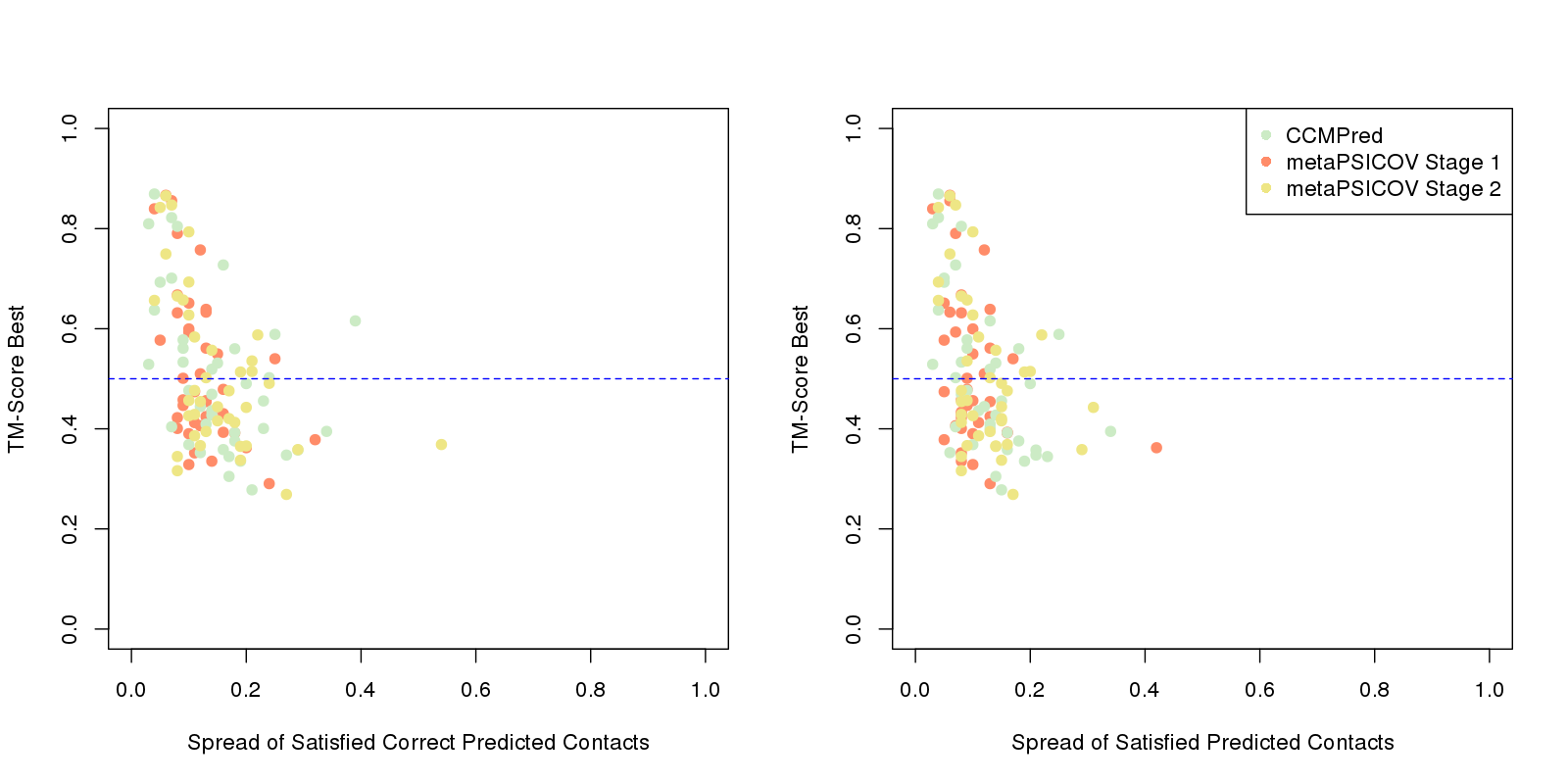

Supplement: Supplementary Data [file btw618_supp.zip › SI_Figure17a.png]

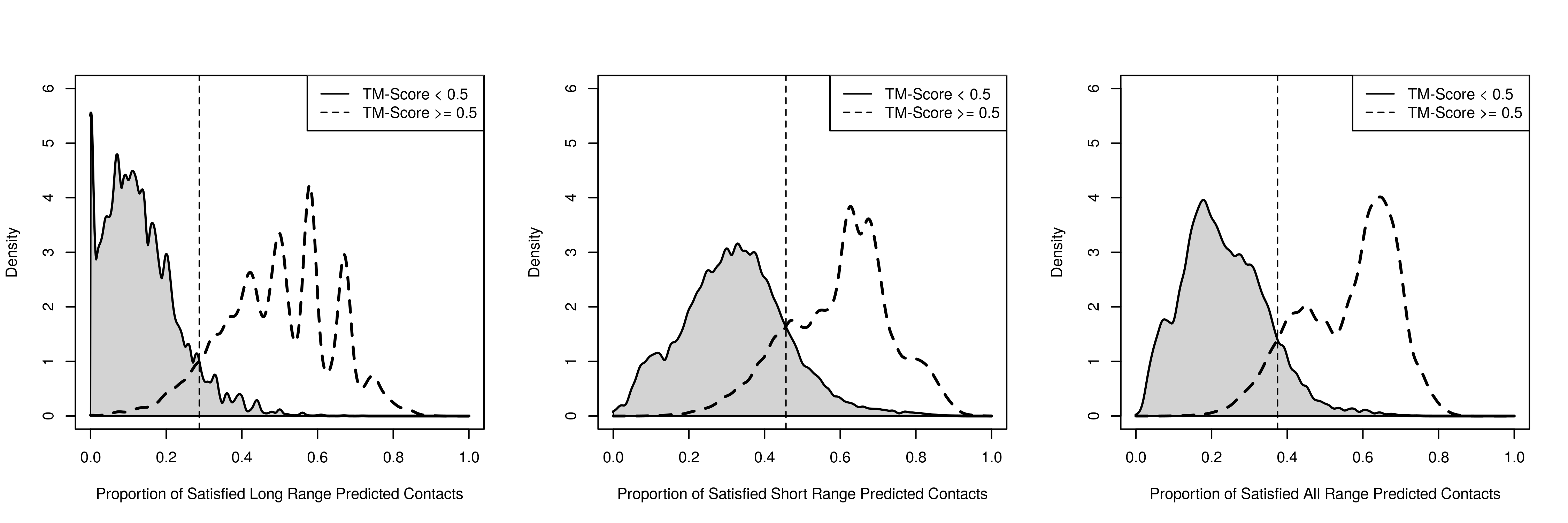

Supplement: Supplementary Data [file btw618_supp.zip › SI_Figure18a.png]

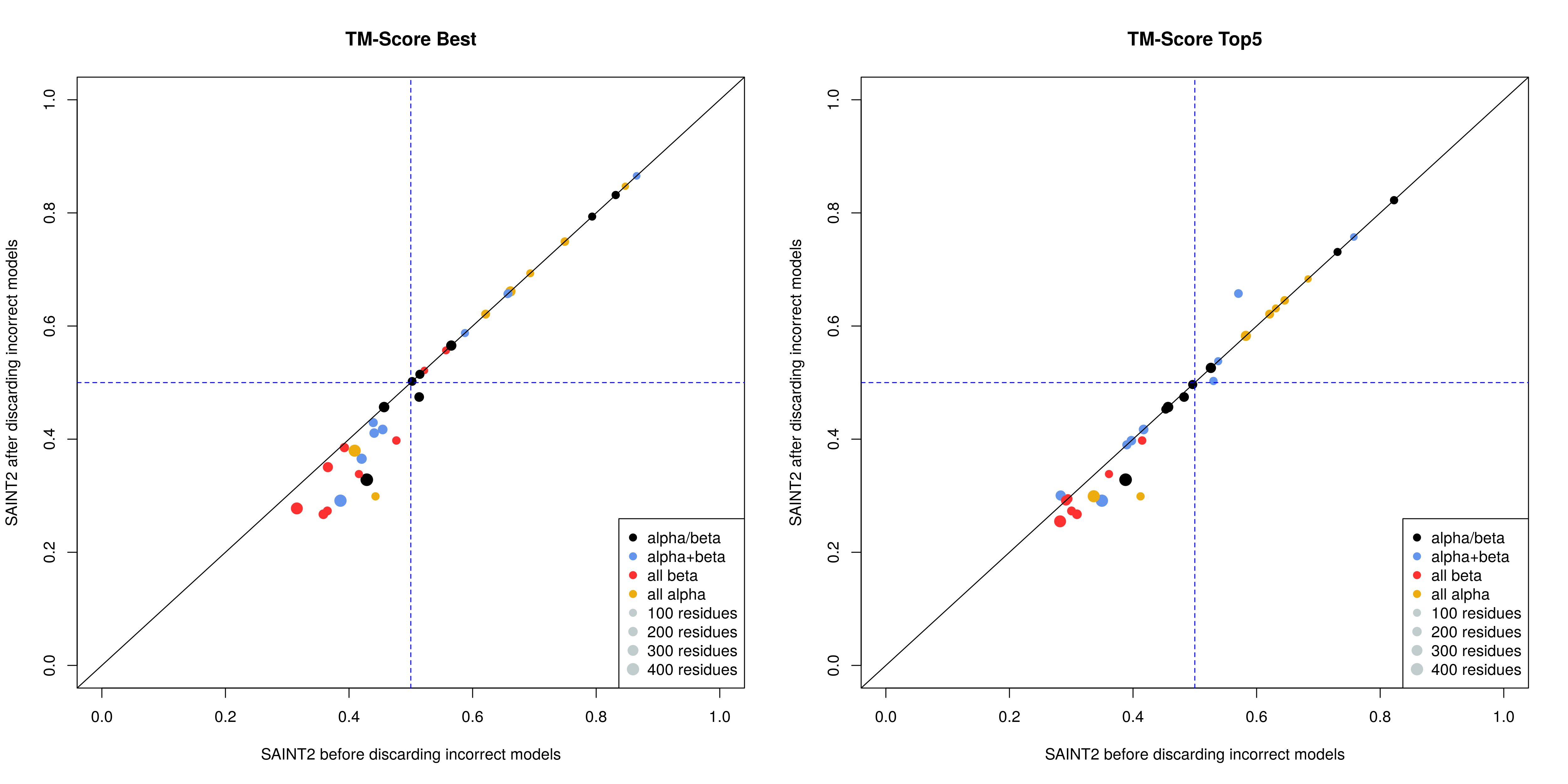

Supplement: Supplementary Data [file btw618_supp.zip › SI_Figure19.png]

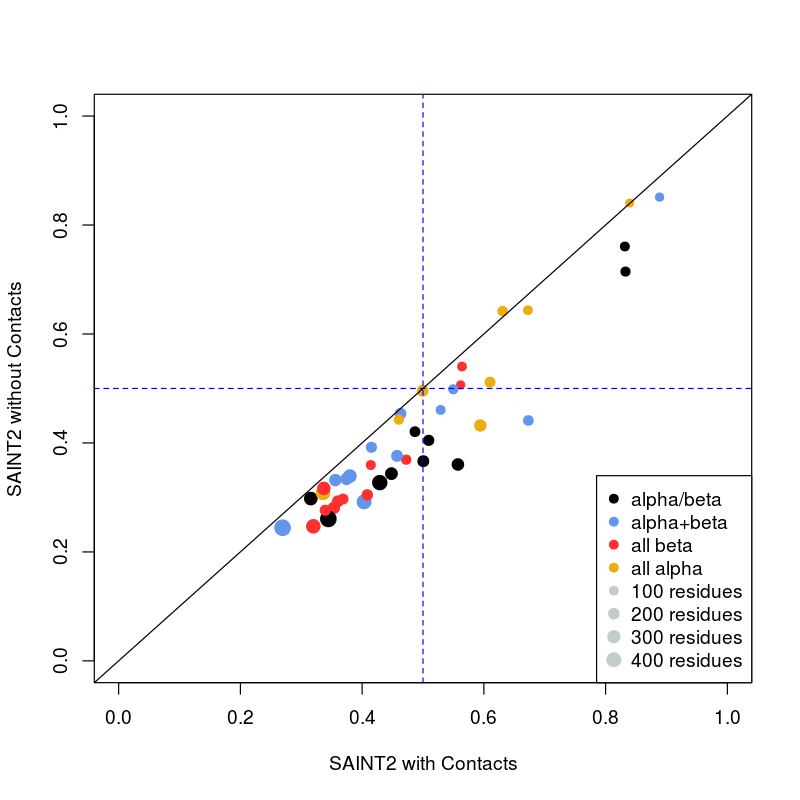

Supplement: Supplementary Data [file btw618_supp.zip › SI_Figure20.png]

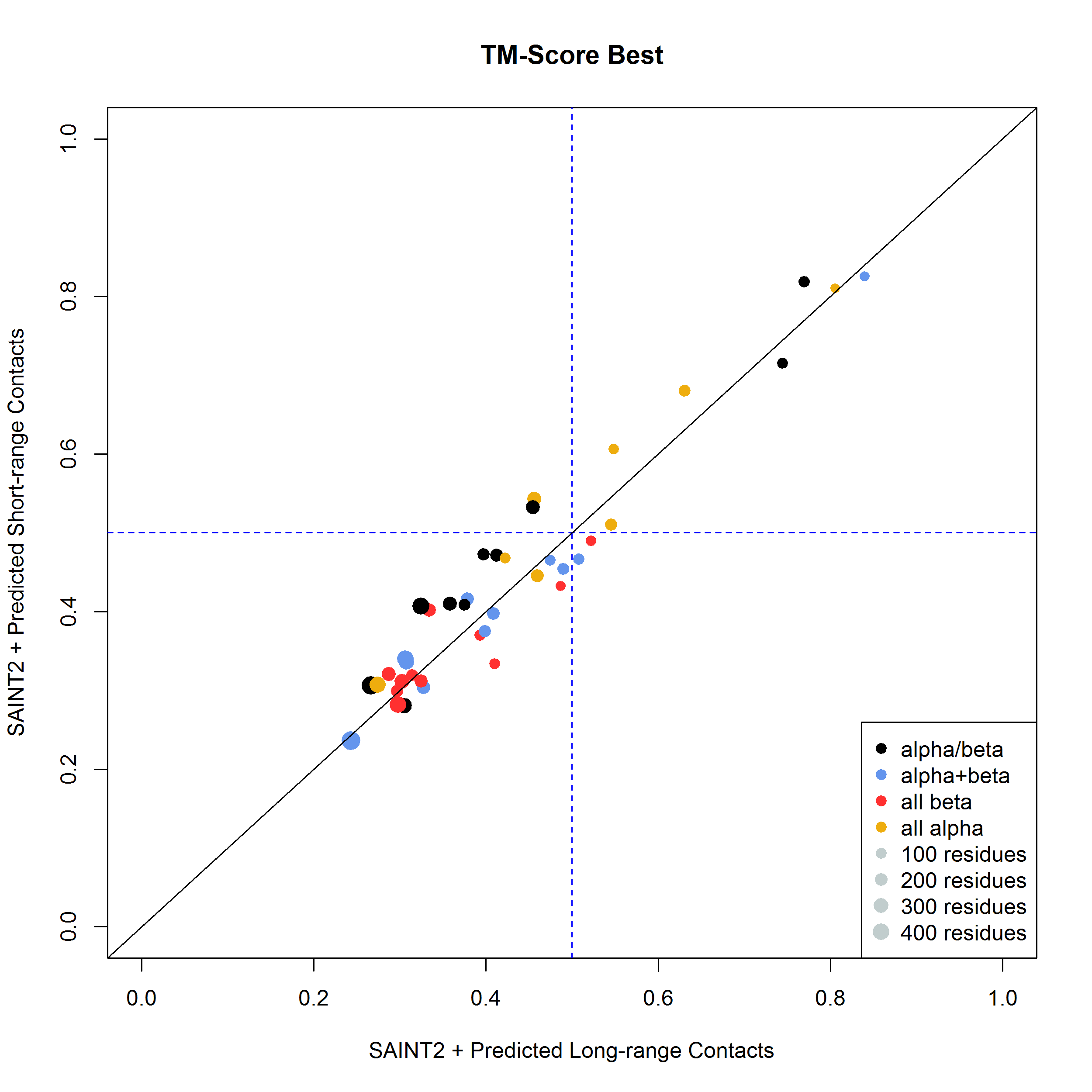

Supplement: Supplementary Data [file btw618_supp.zip › SI_Figure21.png]

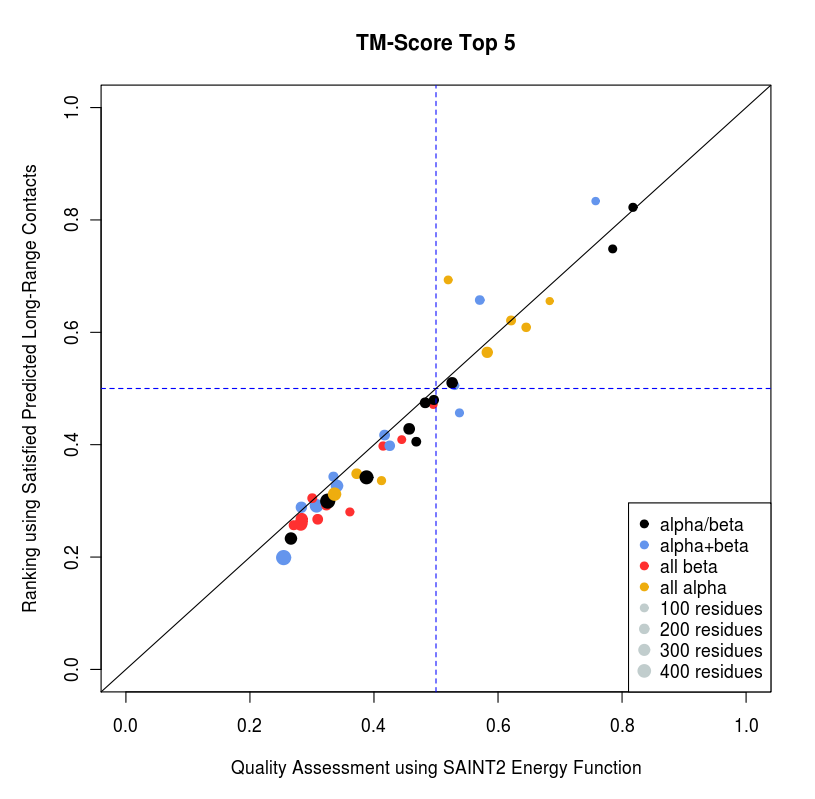

Supplement: Supplementary Data [file btw618_supp.zip › SI_Figure22.png]
